# Extended *N*-Arylsulfonylindoles as 5-HT_6_ Receptor Antagonists: Design, Synthesis & Biological Evaluation

**DOI:** 10.3390/molecules21081070

**Published:** 2016-08-16

**Authors:** Gonzalo Vera, Carlos F. Lagos, Sebastián Almendras, Dan Hebel, Francisco Flores, Gissella Valle-Corvalán, C. David Pessoa-Mahana, Jaime Mella-Raipán, Rodrigo Montecinos, Gonzalo Recabarren-Gajardo

**Affiliations:** 1 Departamento de Farmacia, Facultad de Química, Pontificia Universidad Católica de Chile, Casilla 306, Avda. Vicuña Mackenna 4860, Macul 7820436, Santiago, Chile; glvera@uc.cl (G.V.); sialmend@uc.cl (S.A.); dnhebel@uc.cl (D.H.); fjflores1@uc.cl (F.F.); cpessoa@uc.cl (C.D.P-M.); 2 Department of Endocrinology, School of Medicine, Pontificia Universidad Católica de Chile, Lira 85, 5th Floor, Santiago 8330074, Chile; cflagos@uc.cl; 3 Facultad de Ciencia, Universidad San Sebastián, Lota 2465, Providencia 7510157, Santiago, Chile; 4 Instituto de Química y Bioquímica, Facultad de Ciencias, Universidad de Valparaíso, Casilla 5030, Av. Gran Bretaña 1111, Playa Ancha, Valparaíso 2360102, Chile; gissella.valle@alumnos.uv.cl (G.V.-C.); jaime.mella@uv.cl (J.M.-R.); 5 Departamento de Química-Física, Facultad de Química, Pontificia Universidad Católica de Chile, Casilla 306, Avda. Vicuña Mackenna 4860, Macul 7820436, Santiago, Chile; rmontecinoe@uc.cl

**Keywords:** arylsulfonylindole, 5-HT_6_ receptor antagonists, binding affinity, arylpiperazines

## Abstract

Based on a known pharmacophore model for 5-HT_6_ receptor antagonists, a series of novel extended derivatives of the *N*-arylsulfonyindole scaffold were designed and identified as a new class of 5-HT_6_ receptor modulators. Eight of the compounds exhibited moderate to high binding affinities and displayed antagonist profile in 5-HT_6_ receptor functional assays. Compounds 2-(4-(2-methoxyphenyl)piperazin-1-yl)-1-(1-tosyl-1*H*-indol-3-yl)ethanol (**4b**), 1-(1-(4-iodophenylsulfonyl)-1*H*-indol-3-yl)-2-(4-(2-methoxyphenyl)piperazin-1-yl)ethanol (**4g**) and 2-(4-(2-methoxyphenyl)piperazin-1-yl)-1-(1-(naphthalen-1-ylsulfonyl)-1*H*-indol-3-yl)ethanol (**4j**) showed the best binding affinity (**4b** p*K*_i_ = 7.87; **4g** p*K*_i_ = 7.73; **4j** p*K*_i_ = 7.83). Additionally, compound **4j** was identified as a highly potent antagonist (IC_50_ = 32 nM) in calcium mobilisation functional assay.

## 1. Introduction

**Figure 1:**
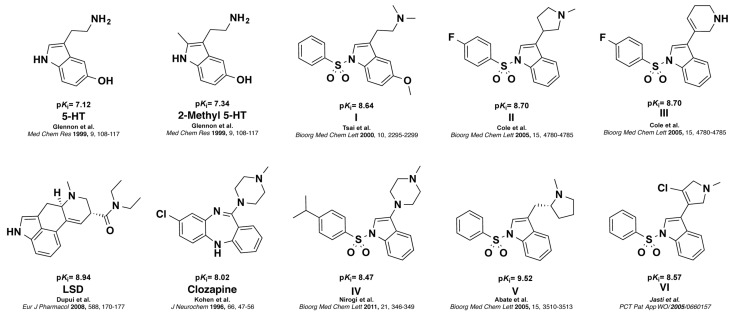
Structure and affinities of 5-HT_6_R ligands. Agonists: 5-HT and 2-Me 5-HT, Antagonists: LSD, Clozapine and *N*-arylsulfonylindole I to VI. (*K*_i_ = inhibitory constant from radioligand binding assays, see Section 4.5).

The human receptor of serotonin (5-HT) subtype 6 (5-HT_6_R), belongs to the class-A seven transmembrane G protein-coupled receptor family [1,2,3]. 5-HT_6_R is positively coupled to Gαs subunits, thereby stimulating adenylate cyclase, and its expression is restricted almost exclusively to limbic and cortical regions of the central nervous system [4]. Evidence that 5-HT_6_R shows high affinity for both typical and atypical antipsychotic drugs, as well as some other psychotropic agents, and that these drugs behave as antagonists of 5-HT_6_R, triggered great interest in establishing the role of 5-HT_6_R in both physiological and pathological conditions [5]. Blockade of the 5-HT_6_R function increases cholinergic and glutamatergic neurotransmission, and improves cognition parameters in a number of animal models of cognitive deficits, suggesting that 5-HT_6_R may have therapeutic utility in the treatment of various neurological and psychiatric disorders such as anxiety, depression and epilepsy [6,7,8]. In addition, previous studies demonstrated that 5-HT_6_R has a major role in obesity, thus boosting the search for novel selective 5-HT_6_R antagonists [9]. 2-Methyl analog of 5-HT was among the first selective 5-HT_6_R agonists reported [10]. LSD and Clozapine are established standard 5-HT_6_R antagonists that have been used as radioligand and control in functional assays, respectively [1,11]. Several potent indole and *N*-arylsulfonylindole derivatives that bind with high affinity to 5-HT_6_R have been also reported in the literature (Figure 1) [12,13,14,15,16].

All reported antagonists I–VI share a common pharmacophore consisting of basic ionizable amine functionality, a sulfonamide moiety as hydrogen bond acceptor group connected to a hydrophobic indole site and an aromatic ring (AR) [17]. Accordingly, a large majority of these compounds are basic. The large majority of these 5-HT_6_R modulators have a basic nitrogen which enables an ionic interaction with residue D106 (3.32). It has not been convincingly demonstrated the need for a basic side chain for effective interaction with the 5-HT_6_ receptor. In this sense, few authors have reported non-basic compounds displaying 5-HT_6_R activity [18,19]. As part of our studies focused on the development of multimodal modulators of serotonergic system, we are interested in evaluating the 5-HT_6_R affinity of less basic analogs of I–VI and exploring the steric limits of a three-dimensional pharmacophore model for 5-HT_6_R antagonists. Herein, we report the design, synthesis, and preliminary pharmacological characterization of two series of weakly basic extended *N*-arylsulfonylindole derivatives targeting 5-HT_6_R.

## 2. Results

### 2.1. Molecular Modeling and Design

**Figure 2:**
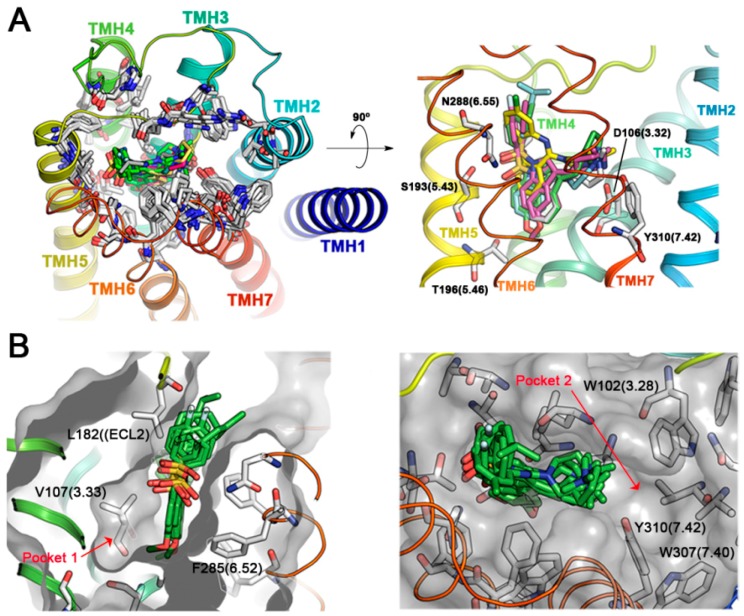
Modeling of 5-HT_6_R interactions with active antagonists. (**A**) Superposition of FRED-predicted binding modes for active antagonists arylsulfonylindoles I–VI and Clozapine in the binding pocket of 5-HT_6_R, delimited by transmembrane helices (TMH) 3–6. Different protein residues conformers and ligands are shown with carbon atoms in white and green color respectively, while Clozapine is shown with yellow carbon atoms (**B**) Alternative hydrophobic pockets in the TMH3-TMH4-TMH5 and TMH2-TMH3-TMH7 regions of the 5-HT_6_R.

Active antagonist ligands I to VI plus Clozapine were docked within the binding site of a model of the human 5-HT_6_R constructed using the human β_2_ adrenergic receptor (β_2_-AR) as a template [20]. As shown in Figure 2A, the predicted binding mode for Clozapine and arylsulfonyl derivatives I–VI occupies the same region within the 5-HT_6_R. The tricyclic system benzene rings of Clozapine which orients perpendicular to the membrane plane, superpose with those from the indole and sulfonyl-attached rings in arylsulfonylindoles derivatives, a binding mode similar to the model proposed by Selent et al. [21]. Arylsulfonylindoles are predicted to bind preferentially within the region delimited by transmembrane helices (TMH) 3–7. Ligands are predicted to bind with the phenyl part of the indole nucleus towards the inner part of the receptor within the hydrophobic pocket delimited by the side-chains of V107 (3.33), A192 (5.42), T196 (5.46), W281 (6.48), and F285 (6.52), whereas the sulfonyl moiety establishes contacts with either S193 (5.43) or N288 (6.55). The aromatic ring pending over the sulfonyl groups is oriented towards the solvent and interacts with residues on the ECL2 such as L182, A184 and other hydrophobic residues such as V189 (5.39) and F284 (6.51). This obtained binding mode, resembles the pharmacophore hypotheses previously proposed by López-Rodríguez et al. [22], but differs from other authors that have reported either an inverse orientation of the indole core for arylsulfonyltryptamines such as the binding mode as reported by Pullagurla et al, or different positioning within the binding pocket such as that reported by Dukat et al. for MS-245 (**I**) [23,24]. The differences may arise from the use of rhodopsin structure as a template for 5-HT_6_R modelling in the latter cases.

**Figure 3:**
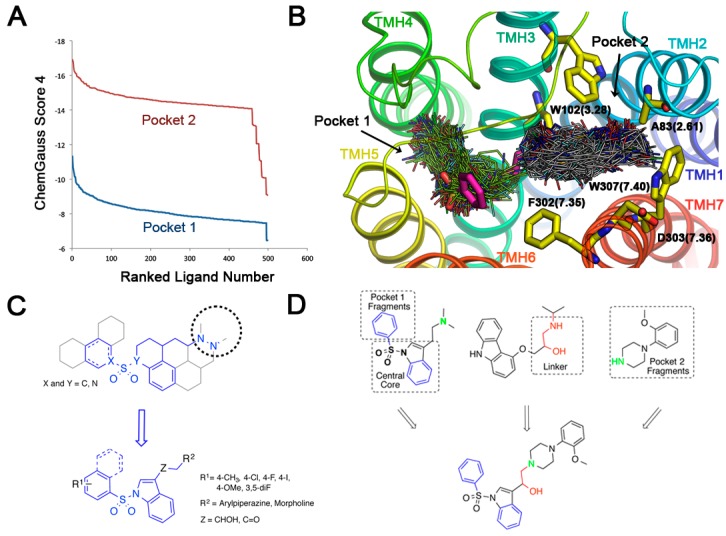
Structure-based design of novel *N*-arylsulfonylindole derivatives targeting the 5-HT_6_R. (**A**) Chemgauss4 scored ranked solution for the Maybridge Ro3 library in pockets 1 and 2 sites in 5-HT_6_R from FRED screening; (**B**) Superposition of FRED-predicted binding modes for active 5-HT_6_R antagonist MS-245 (carbon atoms in magenta) and top ranked compounds from the Maybridge Ro3 database; (**C**) Structural pharmacophore framework model and schematic representation of the synthesized target compounds; (**D**) Prototype designed ligands using a fragment-based strategy in the context of receptor structure, which satisfy the 5-HT_6_R antagonists pharmacophore framework model.

In our 5-HT_6_R model, the predicted binding mode of the active arylsulfonylindoles highlights two additional pockets that were explored in search for putative fragments (Figure 2B). Docking of the Maybridge rule of 3 (Ro3) fragments database (2500 diverse compounds) was performed and the top 500 scored molecules were selected for further analysis. Pocket 1 was explored, and the Chemgauss4 scores ranged from −11.33 to −6.45 for the top 500 scored compounds (Figure 3A). The Chemgauss4 scores ranged from −16.92 to −9.08 for the top 500 scored compounds on pocket 2 allowing us to unsurprisingly identify arylpiperazines and morpholine as putative fragments that might fill a secondary hydrophobic pocket delimited by TMHs 2, 3 and 7 (Figure 3B), where they are predicted to interact with residues A83(2.61), W102(3.28), F302(7.35), D303(7.36) and W307(7.40). Therefore, we reasoned that extending the classic *N*-arylsulfonylindole nucleus with these moieties could provide novel 5-HT_6_R modulators with high binding affinity. The design of the compounds was aimed at exploring the limits of the structural pharmacophore framework model already proposed for *N*-arylsulfonylindole and other classes of 5-HT_6_R ligands (Figure 3C) [25,26]. Superposition of docking positions obtained for the central core indole sulfonamide and fragments for pockets 1 and 2 were then superimposed to determine the common fragments and tolerant regions allowing for merging the chemical moieties (Figure 3D). Superposition of fragment docking results and the structure of carazolol present in the β2-adrenergic receptor template also suggest that the linker present can be used to connect both fragments. Interestingly, the close β-blocker propanolol and pindolol display antagonist activity on other 5-HT receptors [27,28].

### 2.2. Synthesis

**Scheme 1:**
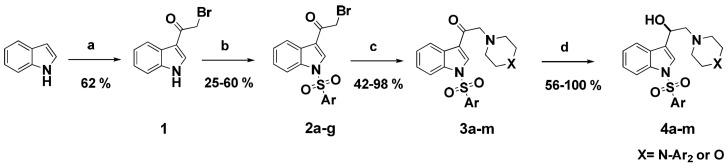
Synthesis of derivatives **3a**–**m** and **4a**–**m**. *Reagents and conditions*: (**a**) (i) anhydrous ZnCl_2_, dry CH_2_Cl_2_, CH_3_MgBr, RT, 2 h; (ii) BrCH_2_COCl, RT, 12 h. (**b**) ArSO_2_Cl, Et_3_N, DMAP, CH_2_Cl_2_, RT, 5 h. (**c**) arylpiperazines or morpholine, K_2_CO_3_, acetone, RT, overnight. (**d**) NaBH_4_, MeOH, RT, 3 h.

Synthesis of the novel series of ligands (**3a**–**m** and **4a**–**m**) was performed as summarized in Scheme 1. We first synthesized 3-(2-bromoacetyl)indole **1** according to the method reported by Bergman and Yang [29,30]. Briefly, acylation was performed in a two-step sequence, starting from a premixed solution of commercial indole and anhydrous zinc chloride, to which was quickly added methyl magnesium bromide to provide the corresponding magnesium salt. This salt undergoes an in situ transmetallation reaction with the zinc chloride previously added. Then, the zinc salt was acylated with bromoacetyl chloride under inert atmosphere to afford bromoacetylindole **1** in a good yield as compared with the literature [29].

The bromoacetylindole **1** previously obtained was further reacted with appropriate aromatic sulfonyl chlorides under basic conditions to afford the corresponding *N*-arylsulfonyl-3-bromoacetylindoles **2a**–**g** with modest to good yields [31].

The prepared haloketones were subjected to bromide displacement in basic medium at room temperature with various arylpiperazines or morpholine to obtain the respective functionalized ethanones **3a**–**m** in very good to excellent yields except compounds **3a** and **3k**, which exhibited a moderate yield (Table 1) [32]. Ketones obtained after the *N*-alkylation reaction above were subsequently reduced with sodium borohydride in methanol to obtain the corresponding alcohols **4a**–**m** (Table 1). The structures of the novel compounds were confirmed through spectroscopic methods. ^1^H-NMR and ^13^C-NMR chemical shifts and physical data are gathered in Section 4.3.

### 2.3. Pharmacology

Synthesized *N*-arylsulfonylindole derivatives were tested in a standard radioligand competition binding assay, using membranes of HEK-293 cells expressing a recombinant human 5-HT_6_ receptor. The compounds were assayed as free bases at eight concentrations in triplicate to obtain the dose-response curves, determine IC_50_ values and calculate *K*_i_ values (Table 2).

All tested compounds displayed inhibition of [^125^I]-SB-258585 binding to 5-HT_6_R [34]. Compounds **4b**, **4f**, **4g**, **4i**, and **4j** were the most potent compounds with p*K*_i_ values of 7.87, 6.43, 7.73, 6.83 and 7.83, respectively. In our hands, the standard 5-HT_6_R antagonist clozapine displayed a p*K*_i_ value of 7.92 (IC_50_ value of 12.4 nM).

The most potent alcohol compounds **4a**, **4b** and **4f**–**4k** from the series were further evaluated for their functional properties in an intracellular calcium mobilization assay (Table 3). The protocol consisted of the addition of test compounds, followed by the addition of known selective 5-HT_6_R agonist 2-Me 5-HT. Upon ligand binding to the receptor, Ca^2+^ is released into the cytoplasm of the cell. A diminished Ca^2+^ mobilization in response to the addition of the test compound would indicate its antagonist activity [35].

All ligands evaluated showed an antagonist profile, since there was no response upon addition of test compounds, but a decrease in the effects following the addition of 2-Me 5-HT. We also determined the IC_50_ value of the standard antagonist clozapine. In this assay the most potent compound was **4j** which had an IC_50_ value of 32 nM.

## 3. Discussion

The vast majority of 5-HT_6_R ligands are highly basic as would be expected for serotonergic ligands [36]. However, it has not been convincingly demonstrated the need for a basic side chain for effective interaction with the 5-HT_6_ receptor. Keeping in mind this issue, we look for new arylsulfonyl derivatives with a less basic character than the ligands already reported. With this aim, we introduced a keto or alcohol group in the linker of new compounds. In the new series synthesized, alcohol derivatives consistently exhibited higher binding affinities when compared to parent keto compounds, except in the pairs of compounds **3c**–**4c**, **3h**–**4h**, and **3l**–**4l**, where *K*_i_ values remained at about the same order of magnitude. The influence of the carbonyl group reduction in receptor binding affinity is remarkable for compounds **4b** (p*K*_i_ = 7.87), **4g** (p*K*_i_ = 7.73) and **4j** (p*K*_i_ = 7.83), for which the affinity increases approximately 100–1000 fold.

The change in affinity might be related to the ability of the compounds to establish an ionic interaction with D106 (Asp3.32), either through a hydrogen bond involving the alcohol group or the protonatable nitrogen atom of the piperazine ring. For example, while the experimental p*K*_a_ value of compound keto **3b** is 5.72, in the alcohol derivative **4g** it is 6.69 (For details see section 4.4), allowing a higher degree of ionized fraction at physiological pH. These p*K*_a_ values are in agreement with those calculated in silico where the keto average p*K*_a_ values are ~5.0 and the alcohol average p*K*_a_ values are near to 7.0. Reduction of the carbonyl group also provides a higher flexibility thus allowing the compounds to be accommodated better within the binding site of the receptor. Moreover, for the hydroxylic compounds series (**4a**–**4m**), the ionized fraction present at physiological pH seems to be a critical property, as the most potent ligands (higher p*K*_i_) have a higher ionized fraction probably owing to the higher p*K*_a_.

**Figure 4:**
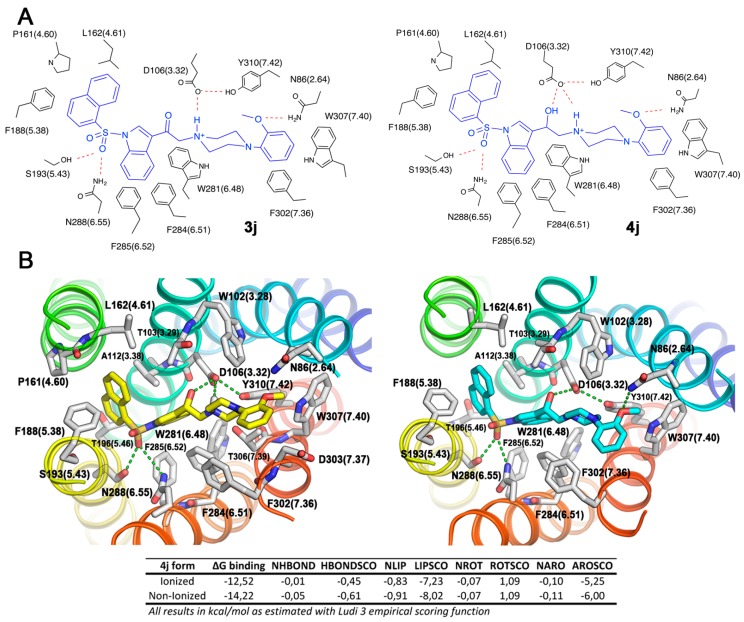
(**A**) 2D schematic representation of the binding mode for compounds **3j** and **4j**, and (**B**) FRED-predicted binding mode for the (*S*) stereoisomer of compound **4j** in its ionized (left) and non-ionized (right) forms within 5-HT_6_R ligand binding site, and the contribution to the binding energy as estimated by the Ludi 3 scoring functions are shown.

We performed docking experiments on the neutral form of the ligands, finding relevant interactions between ligands and the receptor active site. Both the ketone and alcohol derivatives bind with virtually the same set of residues within the orthosteric ligand binding site of 5-HT_6_R (Figure 4A).

The neutral and ionized forms of compound **4j** have similar binding modes (Figure 4B), with the naphthalene ring attached to sulfonyl group occupying the hydrophobic pocket 1 and 2-methoxyphenyl group pending over piperazine ring filling pocket 2. A more stable ion-interaction network accompanied by a small structural reorganization improves the H-bond and aromatic contributions to the binding energy, through 2-methoxyphenyl group H-bond interaction with N86 (3.28) as well as naphthalene aromatic interactions with F188 (5.38). However, the non-ionized form showed a higher aromatic and lipophilic contribution to the binding energy, revealing that an important part of the affinity at the receptor is given by the interaction between this neutral form of the ligands and the active site, a similar finding to that of Harris et al. [37]. Therefore, we propose that ligand biological activity resides in a combination of both protonated and neutral forms.

Following, structural modifications to the aryl group attached to sulfonyl group were explored, showing that electron-withdrawing groups proved detrimental to the binding affinity on the receptor. For instance, when comparing the three 4-haloaryl groups used as substituents in the series of piperazinyl compounds, it can be noted that affinity increases in the order: 4-F (**3e**, p*K*_i_ = 4.72) < 4-Cl (**3d**, p*K*_i_ = 5.07) < 4-I (**3h**, p*K*_i_ = 6.38). Similarly, compounds **3l** and **4l** showed better affinity than the pair **3m**–**4m** (p*K*_i_: 4-OMe-Ph > 3,5-diF-Ph), as previously shown for indole-3-piperazinyl derivatives, for which electron-donating groups in the aryl sulfonamide moiety were preferred for receptor binding instead of halogen atoms [16]. In our study, the 4-iodo substitution represents a special case, given its low electronegativity (comparable to a carbon atom), standing out as one of the best substituents for this series (**4f**–**4h**). In addition, when naphthyl was employed as the aryl group attached to the sulfonyl group, we obtained one of the best binding affinities (**4j**, p*K*_i_ = 7.83), reinforcing the SAR for the aryl substituent at this position on 3-sulfonylindazole derivatives reported by Liu et al. [38]. Regarding the aryl group pending over piperazine ring the best results were obtained with the 2-methoxyphenyl group, as exemplified with compounds **4b** (p*K*_i_ = 7.87), **4g** (p*K*_i_ = 7.73) and **4j** (p*K*_i_ = 7.83), which represent the lead compounds of this series. When these values are compared with a pyridyl group, a decrease in affinity of about 10–35 times is observed using the latter group (**4a** p*K*_i_ = 6.32; **4f** p*K*_i_ = 6.43; **4i** p*K*_i_ = 6.83). Even more marked is the decrease in affinity when we use pyrimidinyl as the aryl group (**4c** p*K*_i_ = 6.07; **4h** p*K*_i_ = 6.21; **4k** p*K*_i_ = 6.22). It should be noted that the 2-methoxyphenyl piperazine exhibits a substantially lower affinity as compared with the compounds here reported [10,39].

## 4. Materials and Methods

### 4.1. Molecular Modeling and Validation of the 5-HT_6_ Receptor

Human β_2_-AR crystal structure was used as a template to model the human 5-HT_6_ receptor (5-HT_6_R). The selection of this structure as template was based on basic local alignment search tool (BLAST) search results and phylogenetic studies [40,41]. The human 5-HT_6_R shares 31.1% sequence identity and 53.9% sequence similarity with human β_2_-AR. The sequence alignment was analyzed to check the preservation of conserved residues throughout the alignment and motifs.

**Figure 5:**
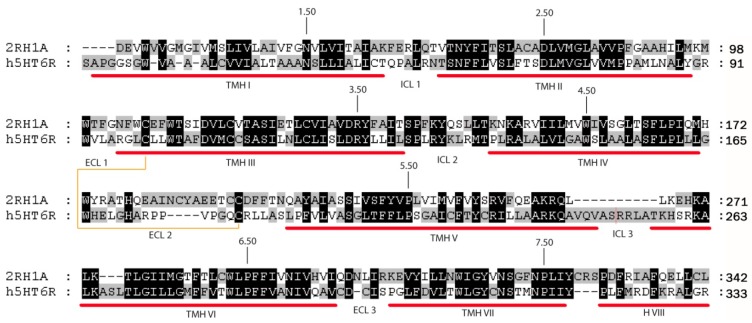
Sequence alignment between 5-HT_6_R and β2-AR. Red bars indicate transmembrane helical regions (TMH) and the brown line connects the residues forming the disulfide bridge. The most conserved residues on each TMH are denoted with the Ballesteros-Weinstein nomenclature [42].

Multiple sequence alignment (Figure 5) and 5-HT_6_R model construction was performed using Modeller as implemented in Discovery Studio (DS) v2.1 (Accelrys Inc., San Diego, CA, USA). Figure 5 shows the final alignment used to generate a set of 100 models, of which the best model according to the internal scoring function of the program (PDF score) was subjected to an energy minimization protocol in order to relax the structure and optimize bond geometry.

**Figure 6:**
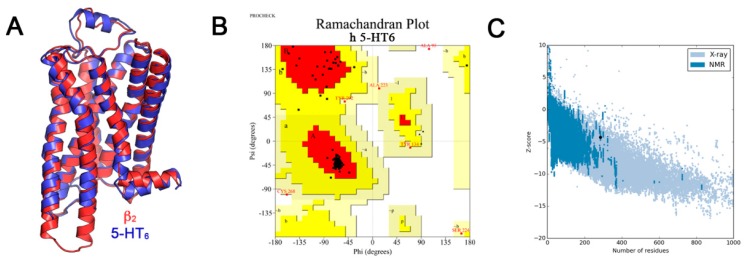
Validation of the obtained 5-HT_6_R model. (**A**) Superposition of the obtained 5-HT_6_R model (in red) and the β_2_-AR structural template (in blue); (**B**) Ramachandran plot for the final minimized 5-HT_6_R model, red color indicates the most favored regions, yellow color for additional allowed regions, light yellow shows generously allowed regions and white color denotes the disallowed regions; (**C**) ProSA-web Z-score plot for 5-HT_6_R model.

A 5000-step steepest descent minimization followed by the conjugate gradient minimization algorithm over 10,000 steps or until the energy decrease between steps became less than 1.0^−5^ kcal/mol was used. The minimization protocol was carried out in a vacuum using a dielectric constant of 4 in order to mimic membrane environment, with a 14 Å cut-off for non-bonded interactions, and the CHARMm force field [43]. The obtained 5-HT_6_R model contains residues 21–305 and a disulfide bridge between residues Cys99 and Cys180. The resulting model was superimposed with the template β2-AR and a root-mean-square deviation (RMSD) of 0.8 Å based on alpha carbon atoms (Cα-RMSD) of 212 equivalent residues (Figure 6) was found. Ramachandran plot analysis with PROCHECK as implemented in NIH SAVES server (http://nihserver.mbi.ucla.edu/SAVES/) [44], shows that the model has more than 98% of the residues in the allowed regions (94.8% in the most favored regions, 2.8% in the additional allowed regions and 1.6% in generously allowed regions). Only two residues (0.8%) fall into the disallowed regions (Figure 6). To evaluate the reliability of the 5-HT_6_R model structure, we used the ProSA-web server which set a Z-score of −4.3 for the human 5-HT_6_R model structure [45]. This value is consistent with the Z-score distribution of experimentally determined structures in the PDB [46].

### 4.2. Receptor-Ligand Interaction Studies

Molecular docking of the Maybridge rule of three fragment database (2500 fragments) and synthesized compounds was performed using FRED v3.0.1 (OpenEye Scientific Software, Santa Fe, NM, USA) [47,48,49]. The binding sites in the 5-HT_6_R were defined and prepared using the FRED receptor GUI, considering the residues involved in the interaction with the 5-HT_6_R binding site as previously defined in the pharmacophore model proposed by Lopez-Rodríguez et al. [22]., and those found in cavity search (Table 4) with Discovery Studio v2.1 (Accelrys Inc.). Pocket 1 and Pocket 2 correspond to Site 3 and 4, respectively. Site 1 corresponds to the whole orthosteric binding pocket in 5-HT_6_R.

Structure canonicalization and salt removal of the Maybridge rule of 3 fragment database (downloadable at www.maybridge.com) was performed using Standardizer v15.12.14 (ChemAxon Ltd., Budapest, Hungary). For the synthesized compounds, ligands were constructed using Marvin Sketch v15.12.14 (ChemAxon Ltd.) and saved as SDF file. Multiconformer libraries of compounds were prepared using OMEGA v2.5.1.4 (OpenEye Scientific Software) [50,51]. QUACPAC v1.6.3.1 (OpenEye Scientific Software) was used to assign the AM1BCC charges to the libraries [52,53,54]. Candidate poses of the ligands within the receptor sites (100) were obtained and optimized using the Chemgauss4 scoring function. Consensus structures of the poses returned from exhaustive docking and optimisation were obtained by consensus scoring using the PLP, Chemscore and Chemgauss3 scoring functions [55]. Finally, the top ranked binding modes for each compound were minimized using the CHARMM22 force field in Discovery Studio v2.1 (Accelrys Inc.). The minimization protocol allowed the side chains of residue within 6 Å from the mass centroid of all docked ligands, using the conjugate gradient algorithm until convergence criteria of 0.001 kcal/mol/Å for the RMS of the energy gradient. Table 5 resumes the energy evaluation performed for each obtained complex using the PLP, LigScore, PMF, and LUDI scoring functions, and the consensus scoring available with Discovery Studio v2.1 (Accelrys Inc.).

### 4.3. Syntheses and Characterization Procedures

All organic solvents used for the synthesis were of analytical grade. All reagents used were purchased from Sigma-Aldrich (St. Louis, MO, USA), Merck (Kenilworth, NJ, USA) or AK Scientific (Union City, CA, USA) and were used as received. Melting points were determined on a Stuart Scientific SMP30 apparatus (Bibby Scientific Limited, Staffordshire, United Kingdom) and are uncorrected. NMR spectra were recorded on a Bruker Avance III HD 400 (Billerica, MA, USA) at 400 MHz for ^1^H and 100 MHz for ^13^C-NMR spectra were recorded in CDCl_3_ unless otherwise indicated, using the solvent signal as reference. The chemical shifts are expressed in ppm (δ scale) downfield from tetramethylsilane (TMS), and coupling constants values (*J*) are given in Hertz. The IR spectra were obtained on a Bruker Vector 22 spectrophotometer (Billerica, MA, USA) using KBr discs. Column chromatography was performed on Merck silica gel 60 (70–230 mesh). Thin layer chromatographic separations were performed on Merck silica gel 60 (70–230 mesh) chromatofoils. Elemental analyses were performed on a FISONS EA 1108 CHNS-O analyzer.

*2-Bromo-1-(1H-indol-3-yl)ethanone* (**1**)


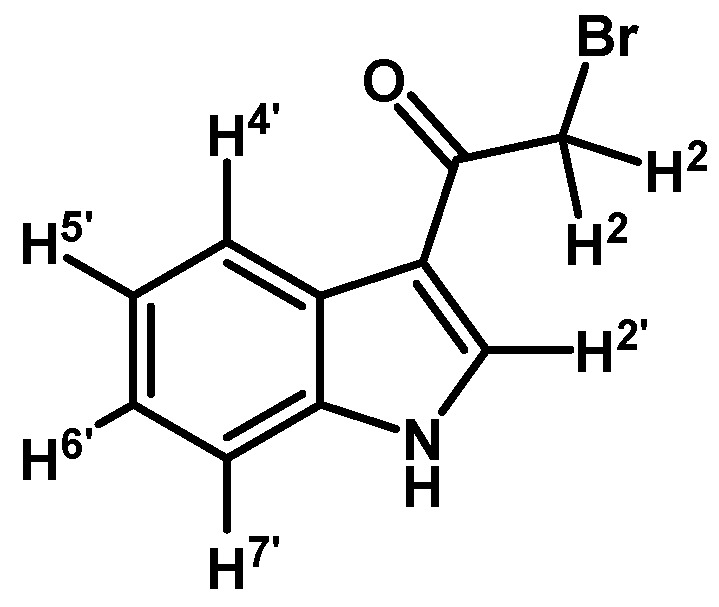


To a solution of indole (1 g, 8.53 mmol) in dry CH_2_Cl_2_ (30 mL) was added anhydrous zinc chloride (1.7 g, 12.45 mmol) under N_2_ atmosphere. Immediately methylmagnesium bromide (6.1 mL, 8.53 mmol, 1.4 M in THF/toluene 1:3) was slowly added over 20 min period and the mixture was vigorously stirred for 2 h at room temperature. After this time, bromoacetyl chloride (0.84 mL, 9.65 mmol) was added in one portion and mixture was stirred until that the starting material had disappeared by checking TLC. The reaction was stopped by dilution adding an aqueous saturated solution of ammonium chloride (50 mL) and extracted with CH_2_Cl_2_ (3 × 30 mL). The combined organic layers were dried with anhydrous sodium sulfate and removal of the solvent under vacuum afforded a residue, which was further purified by column chromatography on silica gel (CH_2_Cl_2_/AcOEt 9:1) to give 1260 mg of (**1**) as a dark red amorphous powder. Yield: 62% m.p.: 160.3–161.0 °C; IR (KBr) cm^−1^: 3210, 1638, 1431, 750. ^1^H-NMR (acetone-d_6_) δ (ppm): 11.21 (s, 1H, N-H), 8.42 (d, *J* = 3.2 Hz, 1H, H-2′), 8.31 (dd, *J* = 7.8 and 4.9 Hz, 1H, H-4′), 7.59–7.53 (m, 1H, H-7′), 7.31–7.24 (m, 2H, H-5′, H-6′), 4.56 (s, 2H, H-2). ^13^C-NMR (acetone-d_6_) δ (ppm): 187.5, 138.3, 135.3, 127.3, 124.7, 123.5, 123.1, 115.6, 113.4, and 33.6. Elemental analysis for C_10_H_8_BrNO (238.08 g/mol) calcd.: C: 50.45; H: 3.39; N: 5.88. Found: C: 50.17; H: 3.73; N: 6.23.

#### 4.3.1. General Procedure for 2-bromo-1-(arylsulfonyl-1*H*-3-yl)ethanone derivatives (**2a**–**g**)

*2-Bromo-1-(1-tosyl-1H-indol-3-yl)ethanone* (**2a**)


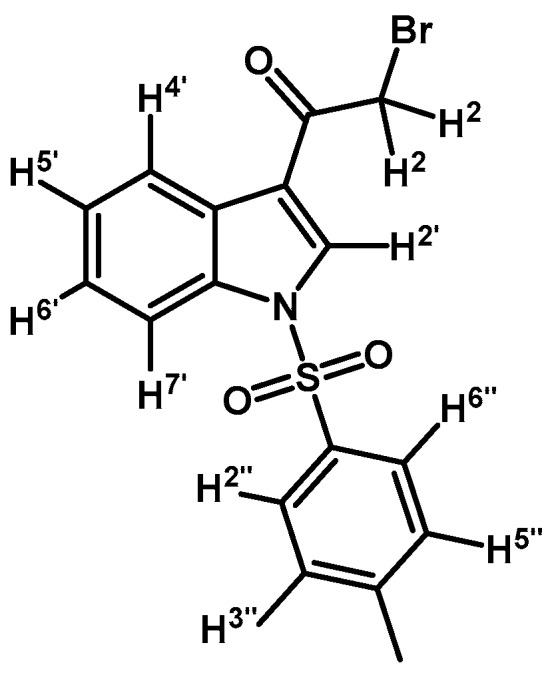


In a round bottom flask under N_2_, 2-bromo-1-(1*H*-indol-3-yl)ethanone (**1**) (500 mg, 2.1 mmol), *p*-toluenesulfonyl chloride (439 mg, 2.3 mmol), DMAP (26 mg, 0.21 mmol), and triethylamine (0.3 mL, 2.1 mmol) were dissolved in 20 mL of dry CH_2_Cl_2_, and the solution was stirred at room temperature until that the starting material had disappeared by checking TLC. The reaction mixture was quenched by dilution with CH_2_Cl_2_ (30 mL), and the organic extract was washed with 1 N HCl (20 mL). The organic layer was dried over anhydrous Na_2_SO_4_, filtered and the solvent was removed under vacuum. The product was purified by silica gel column chromatography using CH_2_Cl_2_ to give 379 mg of (**2a**) as brown crystalline plates. Yield: 46% m.p.: 149.5–150.2 °C; IR (KBr) cm^−1^: 1670, 1536, 1392, 1175, 1137, 993, 749, 569. ^1^H-NMR δ (ppm): 8.34 (s, 1H, H-2′), 8.29 (d, *J* = 7.1 Hz, 1H, H-4′), 7.93 (d, *J* = 7.7 Hz, 1H, H-7′), 7.83 (d, *J* = 8.4 Hz, 2H, H-2′′, H-6′′), 7.42–7.32 (m, 2H, H-5′, H-6′), 7.27 (d, *J* = 8.4 Hz, 2H, H-3′′, H-5′′), 4.59 (s, 2H, H-2), 2.37 (s, 3H, CH_3_). ^13^C-NMR δ (ppm): 187.3, 146.6, 135.1, 134.6, 132.8, 130.8 (2C), 127.9, 127.6 (2C), 126.5, 125.6, 123.2, 118.5, 113.6, 46.5 and 22.1. Elemental analysis for C_17_H_14_BrNO_3_S (392.27 g/mol) calcd.: C: 52.05; H: 3.60; N: 3.57; S: 8.17. Found: C: 51.75; H: 3.97; N: 3.93; S: 8.43.

*2-Bromo-1-(1-(4-chlorophenylsulfonyl)-1H-indol-3-yl)ethanone* (**2b**)


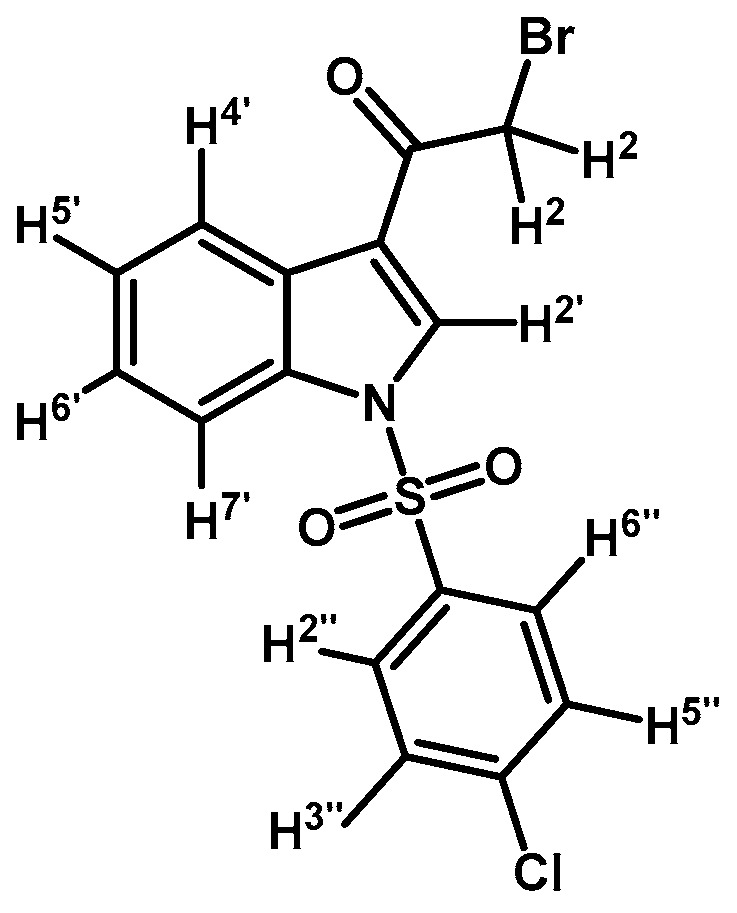


Prepared from 2-bromo-1-(1*H*-indol-3-yl)ethanone (**1**) (500 mg, 2.1 mmol), *p*-chlorobenzenesulfonyl chloride (486 mg, 2.3 mmol), DMAP (26 mg, 0.21 mmol) and triethylamine (0.3 mL, 2.1 mmol) to give a crude, which was purified by column chromatography on silica gel using CH_2_Cl_2_ to afford 520 mg of (**2b**) as brown crystalline plates. Yield: 60% m.p.: 158.1–159.6 °C; IR (KBr) cm^−1^: 1673, 1537, 1387, 1168, 1139, 1083, 998, 756, 569. ^1^H-NMR δ (ppm): 8.24 (d, *J* = 6.0 Hz, 1H, H-4′), 8.23 (s, 1H, H-2′), 7.85 (dd, *J* = 7.0, 1.7 Hz, 1H, H-7′), 7.82 (d, *J* = 8.8 Hz, 2H, H-2′′ and H-6′′), 7.41 (d, *J* = 8.8 Hz, 2H, H-3′′ and H-5′′), 7.37–7.29 (m, 2H, H-5′ and H-6′), 4.50 (s, 2H, H-2). ^13^C-NMR δ (ppm): 187.3, 142.2, 136.0, 135.0, 132.5, 130.5 (2C), 129.0 (2C), 127.9, 126.8, 125.9, 123.6, 119.0, 113.4 and 46.4. Elemental analysis for C_16_H_11_BrClNO_3_S (412.69 g/mol) calcd.: C: 46.57; H: 2.69; N: 3.39; S: 7.77. Found: C: 46.42; H: 3.02; N: 3.54; S: 8.01.

*2-Bromo-1-(1-(4-fluorophenylsulfonyl)-1H-indol-3-yl)ethanone* (**2c**)


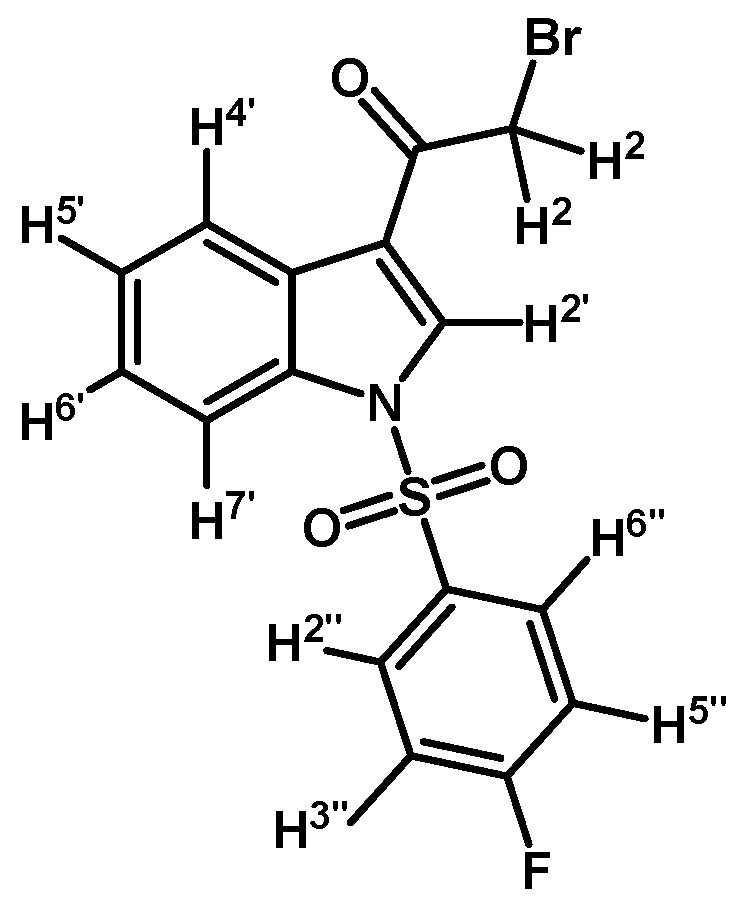


Prepared from 2-bromo-1-(1*H*-indol-3-yl)ethanone (**1**) (500 mg, 2.1 mmol), *p*-fluorobenzenesulfonyl chloride (448 mg, 2.3 mmol), DMAP (26 mg, 0.21 mmol) and triethylamine (0.3 mL, 2.1 mmol) to give a crude, which was purified by column chromatography on silica gel using CH_2_Cl_2_ to give 333 mg of (**2c**) as brown crystalline plates. Yield: 40% m.p.: 137.3–138.6 °C; IR (KBr) cm^−1^: 1676, 1538, 1381, 1167, 1184, 1137, 995, 753, 570. ^1^H-NMR δ (ppm): 8.24 (m, 2H, H-2′ and H-4′), 7.92 (dd, *J* = 9.0 and 4.8 Hz, 2H, H-2′′ and H-6′′), 7.85 (dd, *J* = 7.0 and 1.6 Hz, 1H, H-7′), 7.37–7.29 (m, 2H, H-5′ and H-6′), 7.12 (t, *J* = 8.3 Hz, 2H, H-3′′ and H-5′′), 4.50 (s, 2H, H-2). ^13^C-NMR δ (ppm): 187.3, 166.6 (d, J = 259.2 Hz, 1C), 135.0, 133.7 (d, *J*= 2.7 Hz, 1C), 132.5, 130.6 (d, *J*= 9.9 Hz, 2C), 127.9, 126.8, 125.8, 123.6, 118.9, 117.7 (d, *J*= 23.0 Hz, 2C), 113.4 and 46.4. Elemental analysis for C_16_H_11_BrFNO_3_S (396.23 g/mol) calcd.: C: 48.50; H: 2.80; N: 3.53; S: 8.09. Found: C: 48.23; H: 3.15; N: 3.90; S: 7.79.

*2-Bromo-1-(1-(4-iodophenylsulfonyl)-1H-indol-3-yl)ethanone* (**2d**)


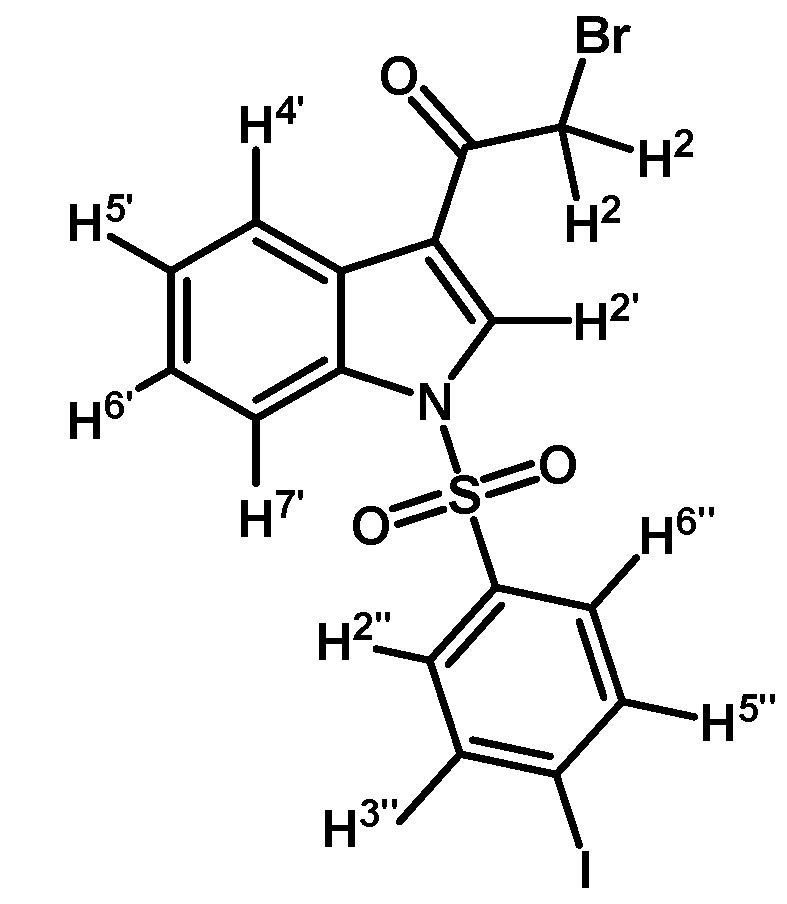


Prepared from 2-bromo-1-(1*H*-indol-3-yl)ethanone (**1**) (1 g, 4.22 mmol), *p*-iodobenzenesulfonyl chloride (1400 mg, 4.64 mmol), DMAP (52 mg, 0.42 mmol) and triethylamine (0.6 mL, 4.2 mmol) to give a crude, which was purified by column chromatography on silica gel using CH_2_Cl_2_ to afford 1142 mg of (**2d**) as brown crystalline plates. Yield: 54% m.p.: 183–184 °C; IR (KBr) cm^−1^: 1673, 1537, 1388, 1167, 1139, 996, 749, 603, 569. ^1^H-NMR δ (ppm): 8.22 (m, 2H, H-2′ and H-4′), 7.82 (d, *J* = 7.4 Hz, 1H, H-7′), 7.75 (d, *J* = 8.6 Hz, 2H, H-3′′ and H-5′′), 7.55 (d, *J* = 8.6 Hz, 2H, H-2” and H-6”), 7.31 (m, 2H, H-5′ and H-6′), 4.50 (s, 2H, H-2). ^13^C-NMR δ (ppm): 187.3, 139.6, 139.3, 137.2 (2C), 135.0 (2C), 133.5, 133.23, 128.6, 127.9, 124.2, 119.0, 103.6, 100.0 and 46.5. Elemental analysis for C_16_H_11_BrINO_3_S (504.14 g/mol) calcd.: C: 38.12; H: 2.20; N: 2.78; S: 6.36. Found: C: 37.78; H: 2.30; N: 3.07; S: 6.59.

*2-Bromo-1-(1-(naphthalen-1-ylsulfonyl)-1H-indol-3-yl)ethanone* (**2e**)


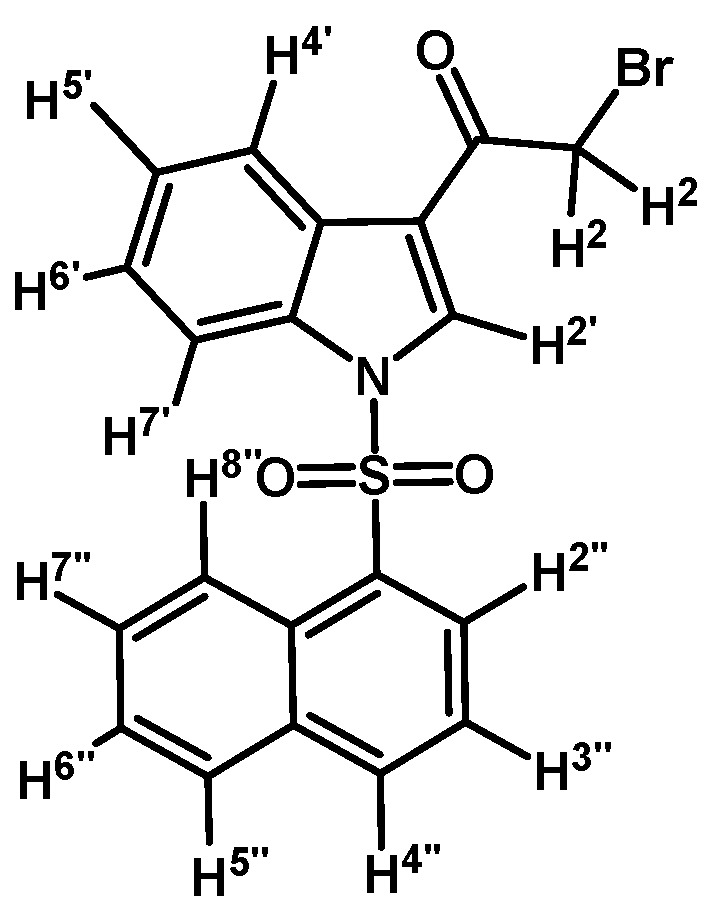


Prepared from 2-bromo-1-(1*H*-indol-3-yl)ethanone (**1**) (320 mg, 1.35 mmol), naphthalenesulfonyl chloride (310 mg, 1.35 mmol), DMAP (17 mg, 0.14 mmol) and triethylamine (0.2 mL, 1.35 mmol) to give a crude, which was purified by column chromatography on silica gel using CH_2_Cl_2_ to afford 147 mg of (**2e**) as brown crystalline plates. Yield: 25% m.p.: 140–141 °C; IR (KBr) cm^−1^: 1678, 1536, 1385, 1164, 1132, 995, 746, 598. ^1^H-NMR δ (ppm): 8.61 (d, *J* = 8.2 Hz, 1H, H-2′′); 8.61 (s, 1H, H-2′); 8.39 (dd, *J* = 0.9 and 7.5 Hz, 1H, H-8′′); 8.23 (dd, *J* = 2.7 and 6.0 Hz, 1H, H-4′); 8.03 (d, *J* = 8.2 Hz, 1H, H-4′′); 7.81 (d, *J* = 8.1 Hz, 1H, H-5′′); 7.73 (dd, *J* = 2.4 and 6.4 Hz, 1H, H-7′); 7.62 (m, 1H, H-3′′); 7.46–7.56 (m, 2H, H-6′′ and H-7′′); 7.26 (m, 2H, H-5′ and H-6′); 4.59 (s, 2H, H-2). ^13^C-NMR δ (ppm): 187.4, 137.1, 135.0, 134.7, 133.4, 132.5, 131.2, 130.0, 129.8, 128.2, 128.0, 127.7, 126.5, 125.5, 124.6, 123.5, 123.4, 117.9, 113.4 and 46.5. Elemental analysis for C_20_H_14_BrNO_3_S (428.30 g/mol) calcd.: C: 56.09; H: 3.29; N: 3.27; S: 7.49. Found: C: 55.82; H: 3.59; N: 3.44; S: 7.88.

*2-Bromo-1-(1-(4-methoxyphenylsulfonyl)-1H-indol-3-yl)ethanone* (**2f**)


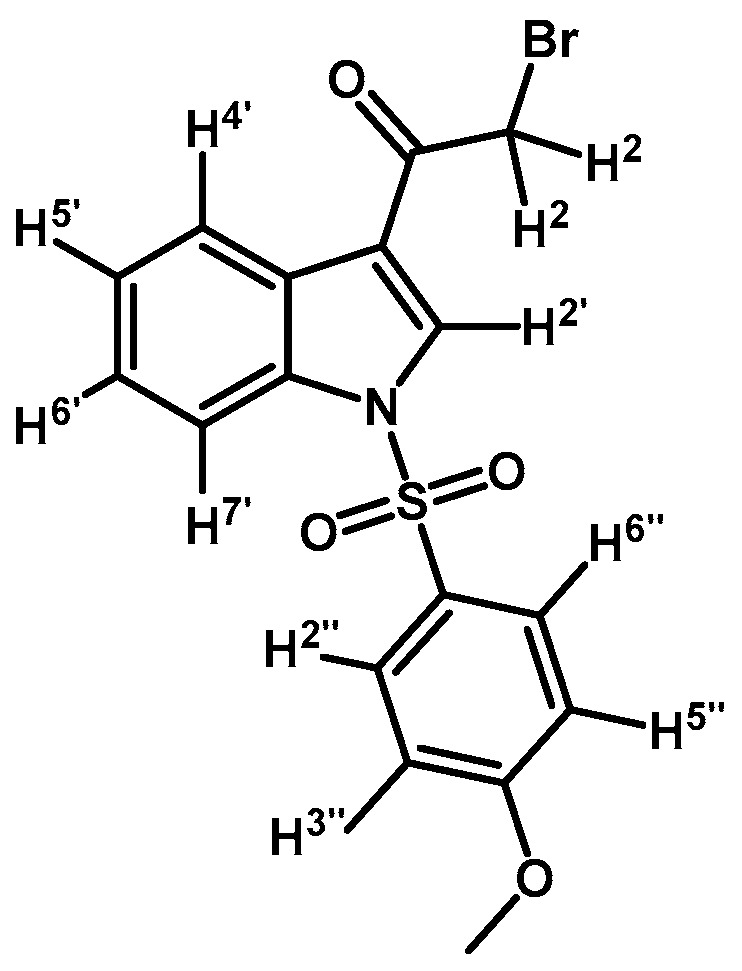


Prepared from 2-bromo-1-(1*H*-indol-3-yl)ethanone (**1**) (894 mg, 3.77 mmol), *p*-methoxybenzenesulfonyl chloride (800 mg, 3.77 mmol), DMAP (52 mg, 0.42 mmol) and triethylamine (0.63 mL, 4.52 mmol) to give a crude, which was purified by column chromatography on silica gel using CH_2_Cl_2_ to afford 1.35 g of (**2f**) as red-brown crystalline plates. Yield: 88% m.p.: 189–190 °C; IR (KBr) cm^−1^: 1672, 1537, 1381, 1170, 1141, 996, 747, 575. ^1^H-NMR δ (ppm): 8.18 (s, 1H, H-2′); 8.15 (d, *J* = 7.8 Hz, 1H, H-4′); 7.78 (d, *J* = 7.8 Hz, 1H, H-7′); 7.75 (d, *J* = 9.0 Hz, 2H, H-2” and H-6”); 7.23 (m, 2H, H-5′ and H-6′); 6.79 (d, *J* = 9.0 Hz, 2H, H-3′′ and H-5′′); 4.43 (s, 2H, H-2); 3.66 (s, 2H, OCH_3_). ^13^C-NMR δ (ppm): 186.9; 164.5; 134.7; 132.4; 129.6 (2C); 128.4; 127.5; 126.1; 125.1; 123.0; 118.0; 114.9 (2C); 113.1; 55.8 and 46.0. Elemental analysis for C_17_H_14_BrNO_4_S (408.27 g/mol) calcd.: C: 50.01; H: 3.46; N: 3.43; S: 7.85. Found: C: 49.85; H: 3.61; N: 3.57; S: 7.59.

*2-Bromo-1-(1-(3,5-difluorophenylsulfonyl)-1H-indol-3-yl)ethanone* (**2g**)


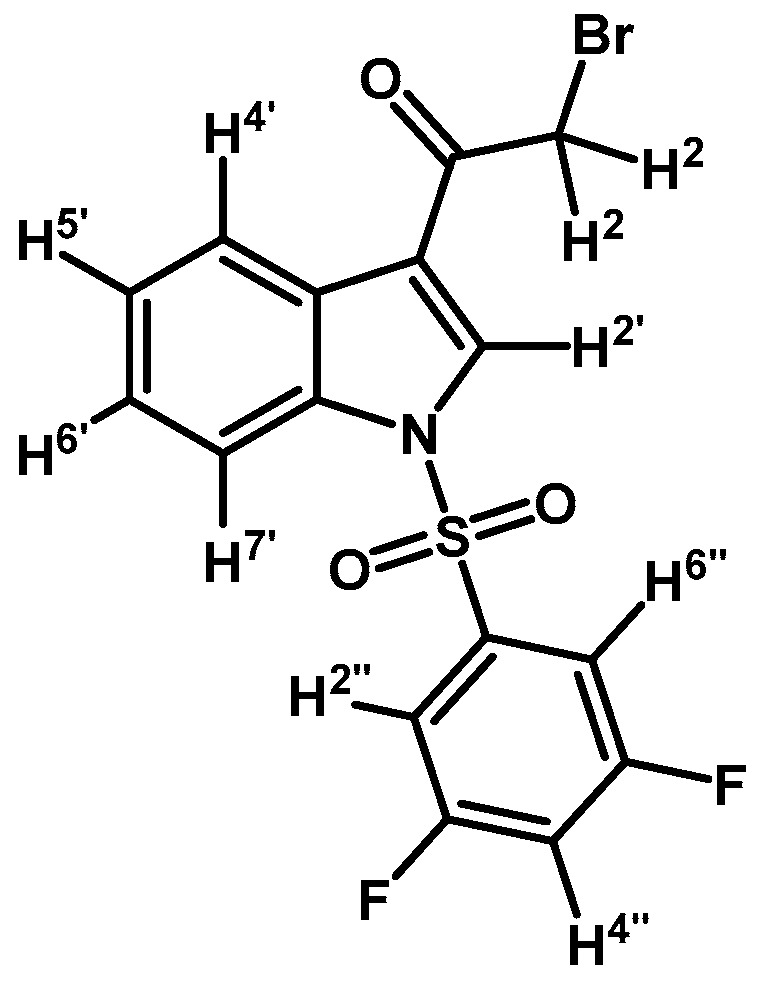


Prepared from 2-bromo-1-(1*H*-indol-3-yl)ethanone (**1**) (1 g, 2.41 mmol), 3,5-difluorobenzenesulfonyl chloride (1.07 g, 5.03 mmol), DMAP (50 mg, 0.41 mmol) and triethylamine (1 mL, 7.0 mmol) to give a crude, which was purified by column chromatography on silica gel using CH_2_Cl_2_ to afford 182 mg of (**2g**) as a pale brown gel. Yield: 18%; m.p.: product in gel state; IR (KBr) cm^−1^: 1692, 1392, 1190, 1134, 1005, 576. ^1^H-NMR δ (ppm): 8.29 (bs, 2H, H-2′ and H-4′); 7.90 (d, *J* = 8.2 Hz, 1H, H-7′); 7.48 (bs, 2H, H-2′′ and H-6′′); 7.41 (m, 2H, H-5′ and H-6′); 7.03 (t, *J* = 8.3 Hz, 1H, H-4′′); 4.60 (s, 2H, H-2). ^13^C-NMR δ (ppm): 187.3;163.3 (dd, *J* = 257.1 and 11.7 Hz, 2C); 140.5 (t, *J* = 8.6 Hz, 1C); 135.0; 132.4; 127.9; 127.1; 126.1; 123.7; 119.5; 113.4; 111.0 (m, 3C); 46.5. Elemental analysis for C_16_H_10_BrF_2_NO_3_S (414.22 g/mol) calcd.: C: 46.39; H: 2.43; N: 3.38; S: 7.74. Found: C: 46.27; H: 2.61; N: 3.53; S: 8.03.

#### 4.3.2. General Procedure for 2-(4-(Aryl)piperazin-1-yl)-1-(1-arylsulfonyl-1*H*-indol-3-yl)ethanone Derivatives (**3a**–**m**)

*2-(4-(Pyridyl-2-yl)piperazin-1-yl)-1-(1-tosyl-1H-indol-3-yl)ethanone* (**3a**)


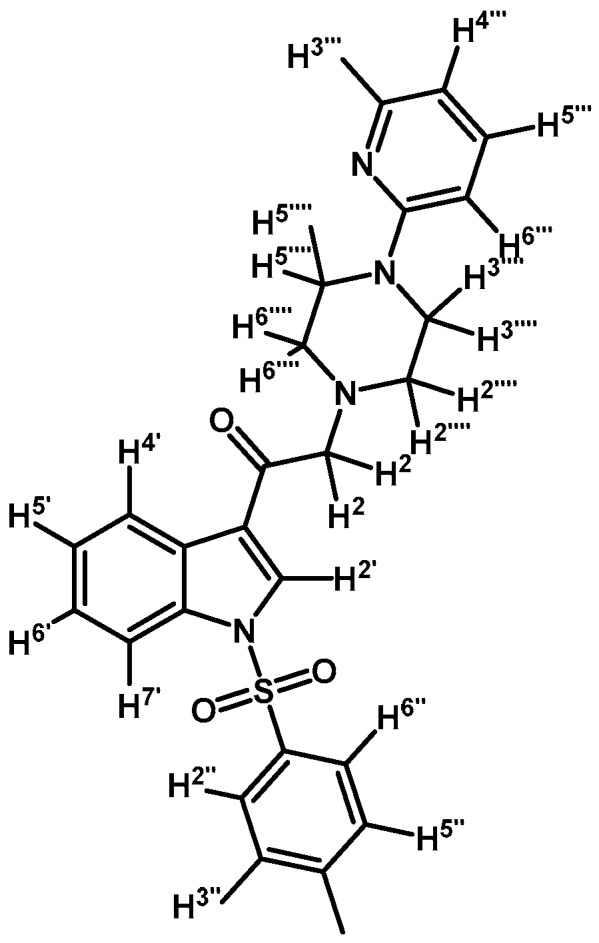


To a solution of 1-(2-pyridyl)-piperazine (125 mg, 0.765 mmol) and potassium carbonate (106 mg, 0.765 mmol) in acetone (30 mL) 2-bromo-1-(1-tosyl-1*H*-indol-3-yl)ethanone (**2a**) (300 mg, 0.765 mmol) was added and the mixture was stirred for 24 h at room temperature. The reaction was stopped by dilution with water (30 mL) and the organic layer was extracted with CH_2_Cl_2_ (3 × 30 mL). The combined organic layers were dried with anhydrous sodium sulfate and removal of the solvent under vacuum afforded a crude residue. The solid was purified by column chromatography on silica gel (CH_2_Cl_2_/acetone 9:1) to yield 165 mg of (**3a**) as orange crystalline plates. Yield: 46% m.p.: 74.9–75.7 °C; IR (KBr) cm^−1^: 1664, 1594, 1437, 1379, 1173, 980, 661. ^1^H-NMR δ (ppm): 8.62 (s, 1H, H-2′), 8.28 (d, *J* = 6.9 Hz, 1H, H-4′), 8.14 (d, *J* = 4.8 Hz, 1H, H-3′′′′), 7.88 (d, *J* = 7.2 Hz, 1H, H-7′), 7.8 (d, *J* = 8.3 Hz, 2H, H-2′′and H-6′′), 7.46–7.39 (m, 1H, H-5′′′′), 7.33–7.24 (m, 2H, H-5′ and H-6′), 7.18 (d, *J* = 7.8 Hz, 2H, H-3′′and H-5′′), 6.62–6.54 (m, 2H, H-4′′′′ and H-6′′′′), 3.64 (s, 2H, H-2), 3.54 (t, *J* = 4.9 Hz, 4H, H-3′′′ and H-5′′′), 2.65 (t, *J* = 4.9 Hz, 4H, H-2′′′ and H-6′′′), 2.27 (s, 3H, CH_3_). ^13^C-NMR δ (ppm): 193.6, 159.9, 148.4, 146.4, 138.0, 134.9, 133.3, 130.7 (2C), 128.6, 128.3, 127.6 (2C), 126.2, 125.3, 123.5, 119.7, 113.9, 113.5, 107.6, 67.0, 53.7 (2C), 45.6 (2C) and 22.0. Elemental analysis for C_26_H_26_N_4_O_3_S (474.57 g/mol) calcd.: C: 65.80; H: 5.52; N: 11.81; S: 6.76. Found: C: 65.94; H: 5.33; N: 11.58; S: 6.63.

*2-(4-(2-Methoxyphenyl)piperazin-1-yl)-1-(1-tosyl-1H-indol-3-yl)ethanone* (**3b**)


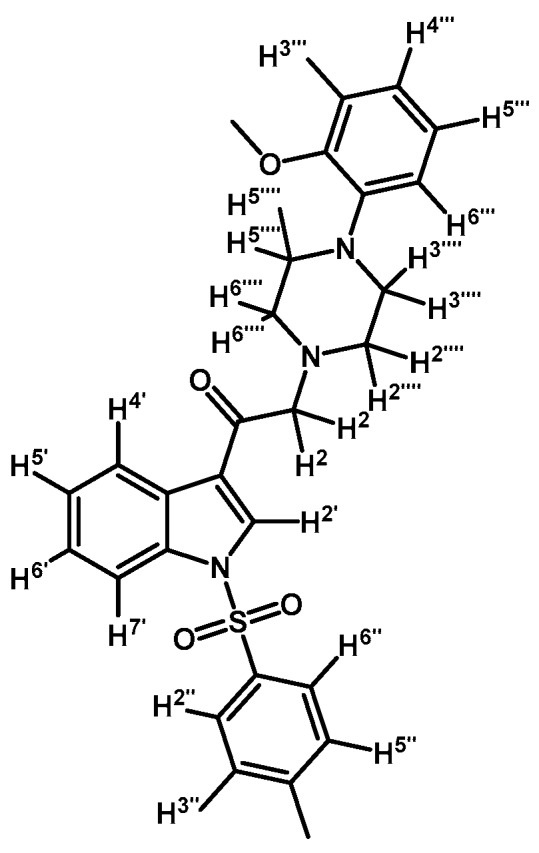


Prepared from 1-(2-methoxyphenyl)-piperazine (147 mg, 0.765 mmol), potassium carbonate (106 mg, 0.765 mmol) and 2-bromo-1-(1-tosyl-1*H*-indol-3-yl)ethanone (**2a**) (300 mg, 0.765 mmol) to give a solid, which was purified by column chromatography on silica gel using CH_2_Cl_2_/acetone 9:1 to obtain 255 mg of pure product (**3b**) as white crystalline plates. Yield: 66% m.p.: 132.3–133.6 °C; IR (KBr) cm^−1^: 1655, 1374 and 1171, 1244, 755, 742. ^1^H-NMR δ (ppm): 8.70 (s, 1H, H-2′), 8.31 (d, *J* = 7.3 Hz, 1H, H-4′), 7.92 (d, *J* = 7.5 Hz, 1H, H-7′), 7.81 (d, *J* = 8.1 Hz, 2H, H-2′′ and H-6′′), 7.36–7.29 (m, 2H, H-5′ and H-6′), 7.22 (d, *J* = 8.1 Hz, 2H, H-3′′ and H-5′′), 7.01–6.90 (m, 3H, H-3′′′, H-4′′′ and H-5′′′), 6.84 (d, *J* = 7.7 Hz, 1H, H-6′′′), 3.84 (s, 3H, O-CH_3_), 3.71 (s, 2H, H-2), 3.13 (bs, 4H, H-3′′′′ and H-5′′′′), 2.80 (bs, 4H, H-2′′′′ and H-6′′′′), 2.31 (s, 3H, CH_3_). ^13^C-NMR δ (ppm): 193.2, 152.3, 145.9, 141.1, 134.6, 134.5, 133.0, 130.3 (2C), 127.9, 127.2 (2C), 125.7, 124.9, 123.1, 123.0, 121.0, 119.3, 118.3, 113.1, 111.3, 66.6, 55.4, 53.7 (2C), 50.5 (2C) and 21.6. Elemental analysis for C_28_H_29_N_3_O_4_S (503.61 g/mol) calcd.: C: 66.78; H: 5.80; N: 8.34; S: 6.37. Found: C: 66.55; H: 5.97; N: 8.21; S: 6.75.

*2-(4-(Pyrimidin-2-yl)piperazin-1-yl)-1-(1-tosyl-1H-indol-3-yl)ethanone* (**3c**)


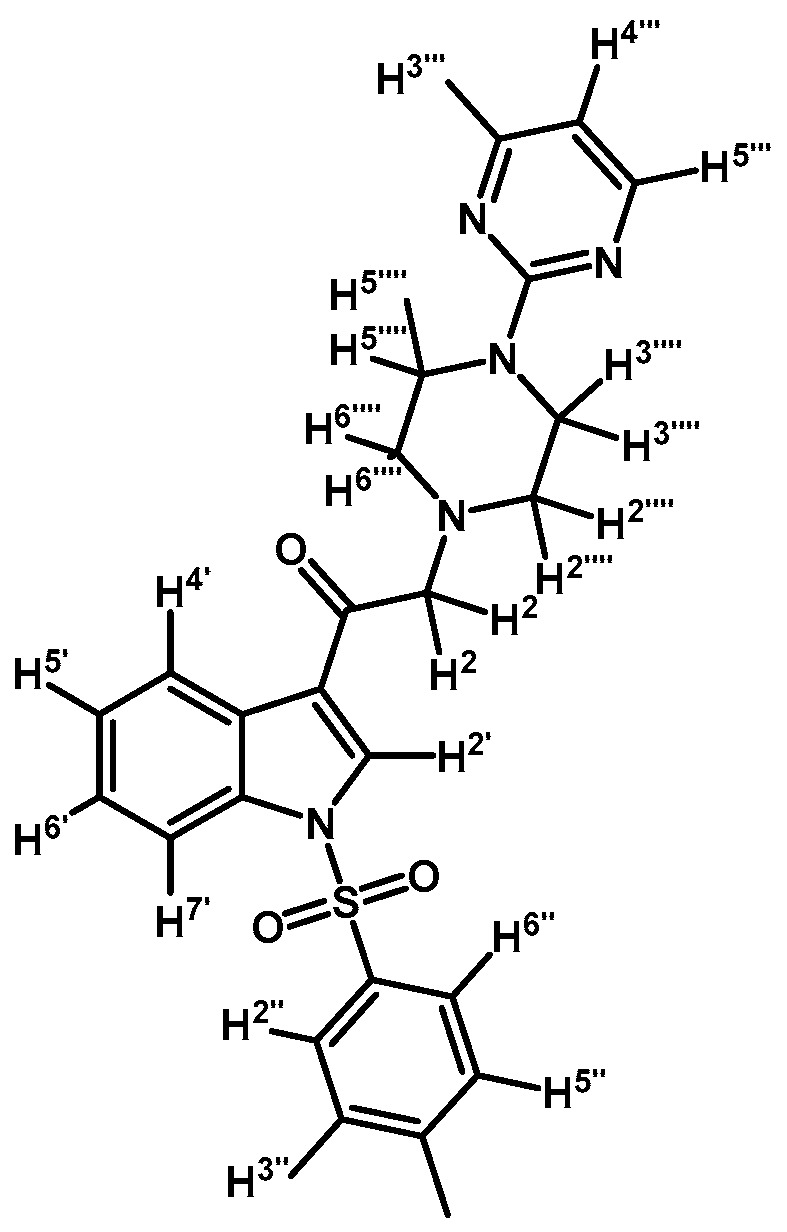


Prepared from 1-(2-pyrimidyl)-piperazine (84 mg, 0.512 mmol), potassium carbonate (70 mg, 0.512 mmol) and 2-bromo-1-(1-tosyl-1*H*-indol-3-yl)ethanone (**2a**) (200 mg, 0.512 mmol) to give a solid, which was purified by column chromatography on silica gel using CH_2_Cl_2_/acetone 9:1 to obtain 221 mg of pure product (**3c**) as white crystalline plates. Yield: 91%; m.p.: 133–134 °C; IR (KBr) cm^−1^: 1651, 1586, 1378, 1173, 571. ^1^H-NMR δ (ppm): 8.72 (s, 1H, H-2′); 8.35 (d, *J* = 7.1 Hz, 1H, H-4′); 8.31 (d, *J* = 4.8 Hz, 2H, H-4′′′ and H-6′′′); 7.95 (d, *J* = 7.6 Hz, 1H, H-7′); 7.84 (d, *J* = 8.3 Hz, 2H, H-2′′ and H-6′′); 7.35 (m, 2H, H-5′ and H-6′); 7.25 (d, *J* = 8.2 Hz, 2H, H-3′′ and H-5′′); 6.49 (t, *J* = 4.7 Hz, 1H, H-5′′′); 3.90 (t, *J* = 4.9 Hz, 4H, H-3′′′′ and H-5′′′′); 3.71 (s, 2H, H-2); 2.66 (t, *J* = 5.0 Hz, 4H, H-2′′′′ and H-6′′′′); 2.33 (s, 3H, CH_3_). ^13^C-NMR δ (ppm): 193.5, 162.0, 158.1 (2C), 146.4, 135.0, 134.9, 133.3, 130.7 (2C), 128.3, 127.5 (2C), 126.1, 125.3, 123.5, 119.8, 113.5, 110.4, 67.0, 53.8 (2C), 44.0 (2C) and 22.0. Elemental analysis for C_25_H_25_N_5_O_3_S (475.56 g/mol) calcd.: C: 63.14; H: 5.30; N: 14.73; S: 6.74. Found: C: 62.75; H: 5.11; N: 14.35; S: 6.66.

*1-(1-(4-Chlorophenylsulfonyl)-1H-indol-3-yl)-2-(4-(pyrimidin-2-yl)piperazin-1-yl)ethanone* (**3d**)


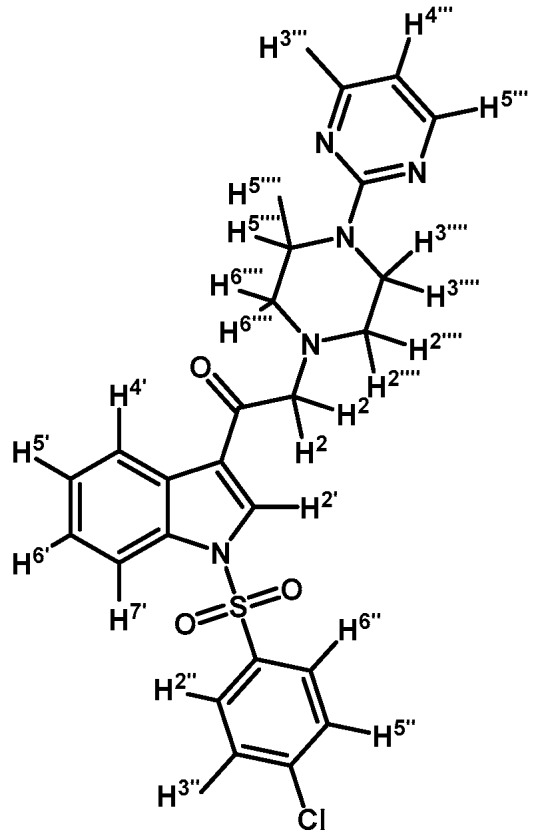


Prepared from 1-(2-pyrimidyl)-piperazine (100 mg, 0.640 mmol), potassium carbonate (90 mg, 0.640 mmol) and 2-bromo-1-(1-(4-chlorophenylsulfonyl)-1*H*-indol-3-yl)ethanone (**2b**) (263 mg, 0.640 mmol) to give a solid, which was purified by column chromatography on silica gel using CH_2_Cl_2_/acetone 4:1 to obtain 281 mg of pure product (**3d**) as white crystalline plates. Yield: 89% m.p.: 168–169 °C; IR (KBr) cm^−1^: 1666, 1587, 1387, 1168, 760, 569. ^1^H-NMR δ (ppm): 8.72 (s, 1H, H-2′); 8.36 (dd, *J* = 6.7 and 2.1 Hz, 1H, H-4′); 8.31 (d, *J* = 4.8, 2H, H-4′′′ and H-6′′′); 7.92 (dd, *J* = 7.0 and 1.6 Hz, 1H, H-7′); 7.87 (d, *J* = 8.7 Hz, 2H, H-2′′ and H-6′′); 7.41 (d, *J* = 8.7 Hz, 2H, H-3′′ and H-5′′); 7.39–7.34 (m, 2H, H-5′ and H-6′); 6.49 (t, *J* = 4.7 Hz, 1H, H-5′′′); 3.91 (t, *J* = 4.9 Hz, 4H, H-3′′′′ and H-5′′′′); 3.72 (s, 2H, H-2); 2.67 (t, *J* = 5.0 Hz, 4H, H-2′′′′ and H-6′′′′). ^13^C-NMR δ (ppm): 193.5, 162.0, 158.1 (2C), 141.8, 136.2, 134.8, 133.0, 130.4 (2C), 128.9 (2C), 128.4, 126.4, 125.6, 123.6, 120.2, 113.3, 110.4, 67.1, 53.8 (2C) and 44.1 (2C). Elemental analysis for C_24_H_22_ClN_5_O_3_S (495.98 g/mol) calcd.: C: 58.12; H: 4.47; N: 14.12; S: 6.46. Found: C: 58.07; H: 4.40; N: 14.27; S: 6.59.

*1-(1-(4-Fluorophenylsulfonyl)-1H-indol-3-yl)-2-(4-(pyrimidin-2-yl)piperazin-1-yl)ethanone* (**3e**)


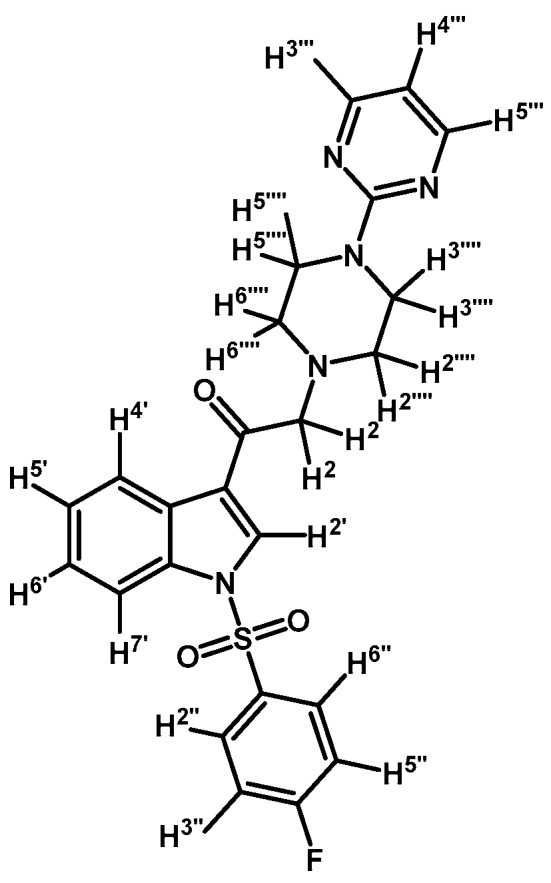


Prepared from 1-(2-pyrimidyl)-piperazine (83 mg, 0.506 mmol), potassium carbonate (70 mg, 0.506 mmol) and 2-bromo-1-(1-(4-fluorophenylsulfonyl)-1*H*-indol-3-yl)ethanone (**2c**) (200 mg, 0.506 mmol) to give a solid, which was purified by column chromatography on silica gel using CH_2_Cl_2_/acetone 9:1 to obtain 187 mg of pure product (**3e**) as white crystalline plates. Yield: 77% m.p.: 145–146 °C; IR (KBr) cm^−1^: 1666, 1587, 1386, 1172, 1186, 571. ^1^H-NMR δ (ppm): 8.71 (s, 1H, H-2′), 8.37 (d, *J* = 7.2 Hz, 1H, H-4′); 8.32 (d, *J* = 4.8 Hz, 2H, H-4′′′ and H-6′′′); 7.98 (dd, *J* = 8.7 and 4.9 Hz, 2H, H-2′′ and H-6′′); 7.93 (d, *J* = 7.6, 1H, H-7′); 7.42–7.33 (m, 2H, H-5′ and H-6′); 7.16 (t, *J* = 8.4 Hz, 2H, H-3′′ and H-5′′); 6.50 (t, *J* = 4.7 Hz, 1H, H-5′′′); 3.91 (t, *J* = 4.9 Hz, 4H, H-3′′′′ and H-5′′′′); 3.72 (s, 2H, H-2); 2.67 (t, *J* = 4.9 Hz, 4H, H-2′′′′ and H-6′′′′). ^13^C-NMR δ (ppm): 193.5, 166.5 (d, *J* = 258.8 Hz, 1C); 162.0, 158.1 (2C), 134.8, 133.9 (d, *J* = 3.2 Hz, 1C), 133.0, 130.4 (d, *J* = 9.8 Hz, 2C), 128.3, 126.4, 125.5, 123.6, 120.1, 117.6 (d, *J* = 23.0 Hz, 2C), 113.3, 110.5, 67.1, 53.8 (2C) and 44.1 (2C). Elemental analysis for C_24_H_22_FN_5_O_3_S (479.53 g/mol) calcd.: C: 60.11; H: 4.62; N: 14.60; S: 6.69. Found: C: 59.92; H: 4.45; N: 14.80; S: 6.83.

*1-(1-(4-Iodophenylsulfonyl)-1H-indol-3-yl)-2-(4-(pyridin-2-yl)piperazin-1-yl)ethanone* (**3f**)


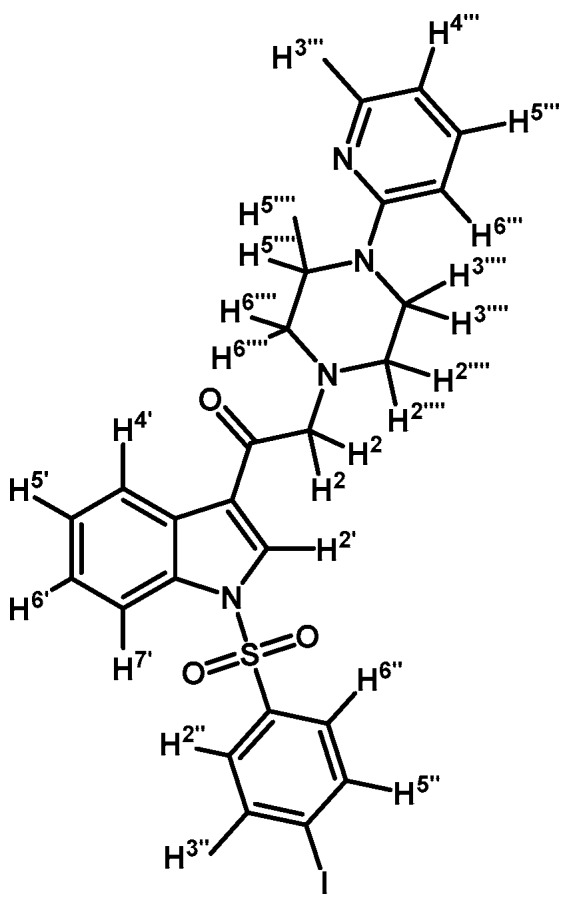


Prepared from 1-(2-pyridyl)-piperazine (97 mg, 0.595 mmol), potassium carbonate (83 mg, 0.595 mmol) and 2-bromo-1-(1-(4-iodophenylsulfonyl)-1*H*-indol-3-yl)ethanone (**2d**) (300 mg, 0.595 mmol) to obtain a crude which was purified by column chromatography employing CH_2_Cl_2_/acetone 9:1 to yield 324 mg of (**3f**) as light yellow crystalline plates. Yield: 93% m.p.: 179–180 °C; IR (KBr) cm^−1^: 1671, 1593, 1479, 1434, 1396, 1163, 1136, 982, 604, 568. ^1^H-NMR δ (ppm): 8.68 (s, 1H, H-2′); 8.36 (dd, *J* = 6.1 and 2.7 Hz, 1H, H-4′); 8.22 (dd, *J* = 4.9 and 1.2 Hz, 1H, H-3′′′); 7.93 (dd, *J* = 6.4 and 2.4, 1H, H-7′); 7.84 (d, *J* = 8.7 Hz, 2H, H-2′′ and H-6′′); 7.64 (d, *J* = 8.7 Hz, 2H, H-3′′ and H-5′′); 7.51 (td, *J* = 8.9, 7.2 and 2.0 Hz, 1H, H-5′′′); 7.35–7.43 (m, 2H, H-5′ and H-6′); 6.63–6.70 (m, 2H, H-4′′′ and H-6′′′); 3.70 (s, 2H, H-2); 3.62 (t, *J* = 5.0 Hz, 4H, H-3′′′′ and H-5′′′′); 2.72 (t, *J* = 5.0 Hz, 4H, H-2′′′′ and H-6′′′′). ^13^C-NMR δ (ppm): 193.5, 159.9, 148.4, 139.4 (2C), 138.0, 137.5, 134.8, 133.0, 128.6 (2C), 128.4, 126.4, 125.6, 123.7, 120.2, 114.0, 113.4, 107.6, 103.2, 67.2, 53.8 (2C) and 45.7 (2C). Elemental analysis for C_25_H_23_IN_4_O_3_S (586.44 g/mol) calcd.: C: 51.20; H: 3.95; N: 9.55; S: 5.47. Found: C: 51.32; H: 3.82; N: 9.92; S: 5.20.

*1-(1-(4-Iodophenylsulfonyl)-1H-indol-3-yl)-2-(4-(2-methoxyphenyl)piperazin-1-yl)ethanone* (**3g**)


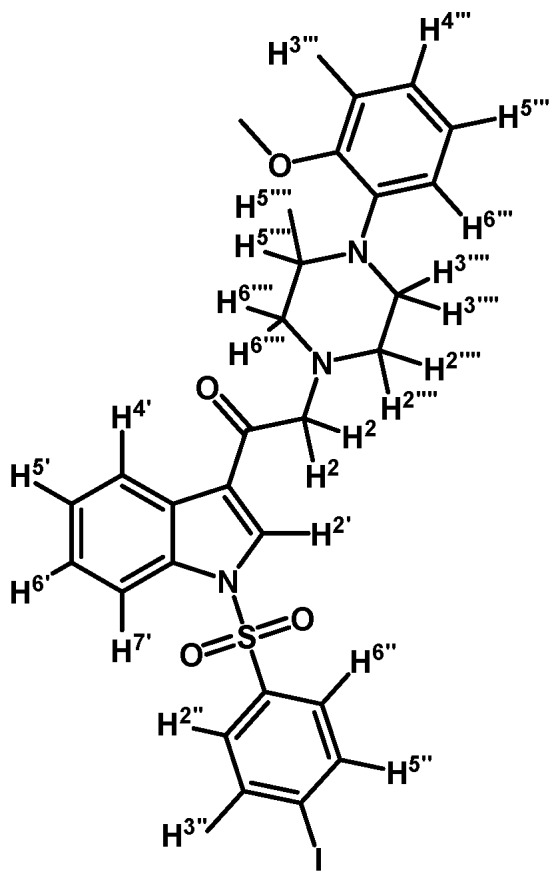


Prepared from 1-(2-methoxyphenyl)-piperazine (114 mg, 0.595 mmol), potassium carbonate (82 mg, 0.595 mmol) and 2-bromo-1-(1-(4-iodophenylsulfonyl)-1*H*-indol-3-yl)ethanone (**2d**) (300 mg, 0.595 mmol) to give a solid, which was purified by column chromatography on silica gel using CH_2_Cl_2_/acetone 9:1 to give 358 mg of (**3g**) as orange crystalline plates. Yield: 98% m.p.: 182–183 °C; IR (KBr) cm^−1^: 1655, 1385, 1172, 741, 603, 570. ^1^H-NMR δ (ppm): 8.72 (s, 1H, H-2′); 8.36 (dd, *J* = 6.4 and 2.6 Hz, 1H, H-4′); 7.93 (dd, *J* = 6.6 and 2.2 Hz, 1H, H-7′); 7.82 (d, *J* = 8.6 Hz, 2H, H-2′′ and H-6′′); 7.64 (d, *J* = 8.6 Hz, 2H, H-3′′ and H-5′′); 7.35–7.41 (m, 2H, H-5′ and H-6′); 7.05–7.00 (m, 1H, H-5′′′); 6.99–6.94 (m, 2H, H-3′′′ and H-4′′′); 6.88 (d, *J* = 7.7 Hz, 1H, H-6′′′); 3.87 (s, 3H, OCH_3_); 3.71 (s, 2H, H-2); 3.15 (bs, 4H, H-3′′′′ and H-5′′′′); 2.81 (bs, 4H, H-2′′′′ and H-6′′′′). ^13^C-NMR δ (ppm): 193.8, 152.7, 139.4 (2C), 137.6, 134.9, 133.2, 128.6 (2C), 128.4, 126.4, 125.6, 123.7, 123.6, 121.5, 120.3, 118.7, 113.4, 111.8, 103.2, 67.3, 55.8, 54.2 (2C) and 51.1 (2C). Elemental analysis for C_27_H_26_IN_3_O_4_S (615.48 g/mol) calcd.: C: 52.69; H: 4.26; N: 6.83; S: 5.21. Found: C: 52.60; H: 4.21; N: 6.80; S: 4.97.

*1-(1-(4-Iodophenylsulfonyl)-1H-indol-3-yl)-2-(4-(pyrimidin-2-yl)piperazin-1-yl)ethanone* (**3h**)


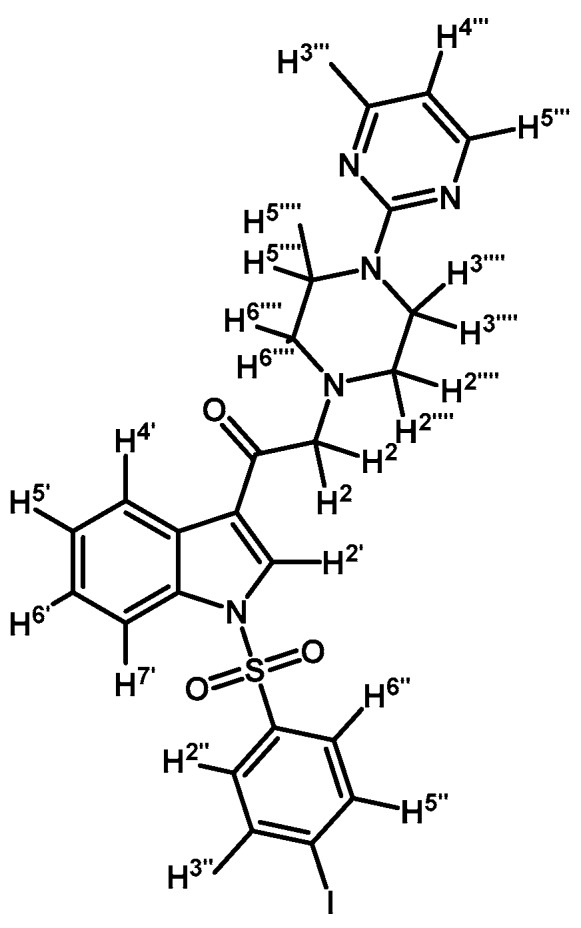


Prepared from 1-(2-pyrimidyl)-piperazine (100 mg, 0.595 mmol), potassium carbonate (82 mg, 0.595 mmol) and 2-bromo-1-(1-(4-iodophenylsulfonyl)-1*H*-indol-3-yl)ethanone (**2d**) (300 mg, 0.595 mmol) to give a solid, which was purified by column chromatography on silica gel using CH_2_Cl_2_/acetone 9:1 to obtain 334 mg of (**3h**) as white crystalline plates. Yield: 96% m.p.: 184–185 °C; IR (KBr) cm^−1^: 1667, 1587, 1386, 1169, 981, 740, 569. ^1^H-NMR δ (ppm): 8.68 (s, 1H, H-2′); 8.36 (dd, *J* = 6.3 and 2.5 Hz, 1H, H-4′); 8.2 (d, *J* =, 4.7 Hz, 2H, H-4′′′ and H-6′′′); 7.91, (dd, *J* = 6.6 and 2.2 Hz, 1H, H-7′); 7.82 (d, *J* = 8.7 Hz, 2H, H-2′′ and H-6′′); 7.63 (d, *J* = 8.7 Hz, 2H, H-3′′ and H-5′′); 7.38 (m, 2H, H-5′ and H-6′); 6.50 (t, *J* = 4.7 Hz, 1H, H-5′′′); 3.91 (t, *J* = 4.9 Hz, 4H, H-3′′′′ and H-5′′′′); 3.70 (s, 2H, H-2); 2.67 (t, *J* = 5.0 Hz, 4H, H-2′′′′ and H-6′′′′). ^13^C-NMR δ (ppm): 193.5, 162.0, 158.2 (2C), 139.4 (2C), 137.5, 134.8, 132.9, 128.6 (2C), 128.4, 126.4, 125.6, 123.7, 120.3, 113.3, 110.5, 103.3, 67.1, 53.8 (2C) and 44.1 (2C). Elemental analysis for C_24_H_22_IN_5_O_3_S (587.43 g/mol) calcd.: C: 49.07; H: 3.77; N: 11.92; S: 5.46. Found: C: 49.11; H: 3.71; N: 12.09; S: 5.50.

*1-(1-(Naphthalen-1-ylsulfonyl)-1H-indol-3-yl)-2-(4-(pyridin-2-yl)piperazin-1-yl)ethanone* (**3i**)


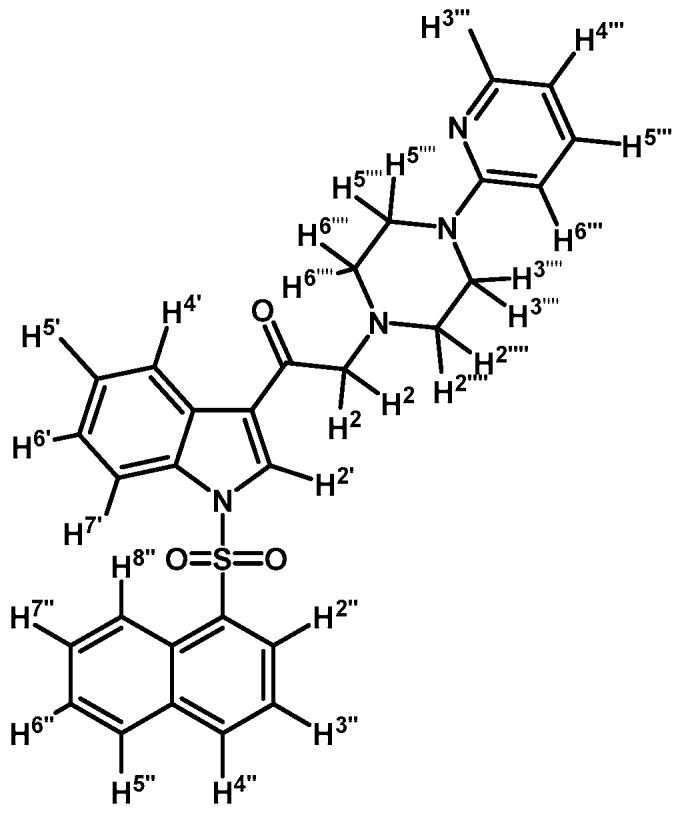


Prepared from 1-(2-pyridyl)-piperazine (27 mg, 0.163 mmol), potassium carbonate (23 mg, 0.163 mmol) and 2-bromo-1-(1-(naphthalen-1-ylsulfonyl)-1*H*-indol-3-yl)ethanone (**2e**) (70 mg, 0.163 mmol) to give a solid, which was purified by column chromatography on silica gel using CH_2_Cl_2_/acetone 9:1 to obtain 71 mg of (**3i**) as white crystalline plates. Yield: 86%; m.p.: 76–77 °C; IR (KBr) cm^−1^: 1664, 1593, 1437, 1371, 1169, 1133, 769. ^1^H-NMR δ (ppm): 8.96 (s, 1H, H-2′); 8.65 (d, *J* = 8.6 Hz, 1H, H-2′′); 8.38 (dd, *J* = 7.5 and 1.0 Hz, 1H, H-8′′); 8.34 (dd, *J* = 6.2 and 2.9 Hz, 1H, H-4′); 8.22 (dd, *J* = 4.8 and 1.0 Hz, 1H, H-3′′′); 8.04 (d, *J* = 8.2 Hz, 1H, H-4′′); 7.84 (d, *J* = 8.1 Hz, 1H, H-5′′); 7.81 (dd, *J* = 6.3 and 3.1 Hz, 1H, H-7′); 7.60 (m, 1H, H-3′′); 7.56 (t, *J* = 7.8 Hz, 1H, H-5′′′); 7.53–747 (m, 2H, H-6′′ and H-7′′); 7.31 (dd, *J* = 6.1 and 3.2 Hz, 2H, H-5′ and H-6′); 6.68–6.62 (m, 2H, H-4′′′ and H-6′′′); 3.69 (s, 2H, H-2); 3.55 (t, *J* = 4.9 Hz, 4H, H-3′′′′ and H-5′′′′); 2.69 (t, *J* = 5.0 Hz, 4H, H-2′′′′ and H-6′′′′). ^13^C-NMR δ (ppm): 193.6, 159.8, 148.3, 138.0, 136.9, 134.9, 134.7, 133.7, 133.0, 130.9, 129.9, 129.7, 128.4, 128.1, 127.9, 126.1, 125.3, 124.6, 123.7, 123.5, 119.3, 114.0, 113.4, 107.6, 67.3, 53.7 (2C) and 45.6 (2C). Elemental analysis for C_29_H_26_N_4_O_3_S (510.61 g/mol) calcd.: C: 68.21; H: 5.13; N: 10.97; S: 6.28. Found: C: 67.89; H: 5.40; N: 11.08; S: 6.48.

*2-(4-(2-Methoxyphenyl)piperazin-1-yl)-1-(1-(naphthalen-1-ylsulfonyl)-1H-indol-3-yl)ethanone* (**3j**)


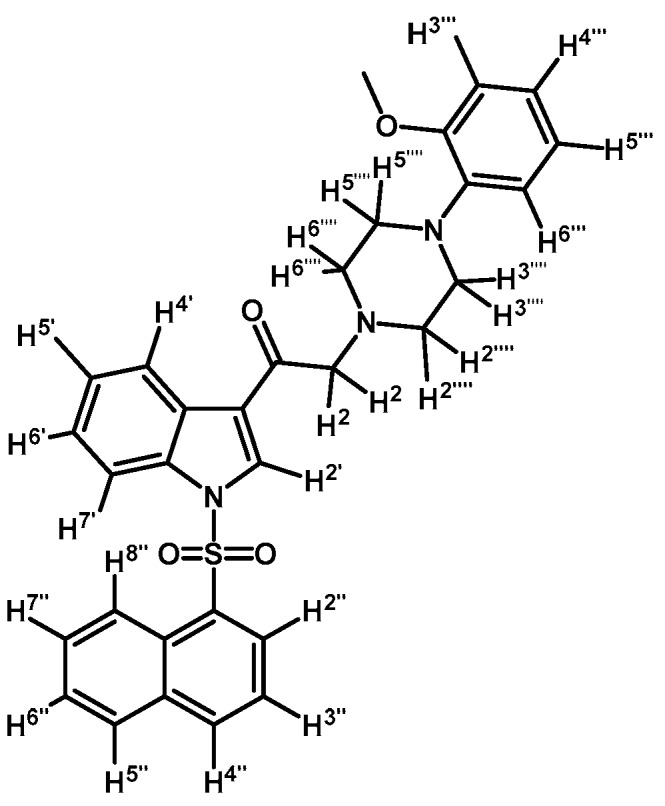


Prepared from 1-(2-methoxyphenyl)-piperazine (31 mg, 0.163 mmol), potassium carbonate (23 mg, 0.163 mmol) and 2-bromo-1-(1-(naphthalen-1-ylsulfonyl)-1*H*-indol-3-yl)ethanone (**2e**) (70 mg, 0.163 mmol) to give a solid, which was purified by column chromatography on silica gel using CH_2_Cl_2_/AcOEt 9:1 to give 65 mg of (**3j**) as white crystalline plates. Yield: 74%; m.p.: 74–75 °C; IR (KBr) cm^−1^: 1663, 1372, 1172, 1133, 745. ^1^H-NMR δ (ppm): 8.99 (s, 1H, H-2′); 8.67 (d, *J* = 8.7 Hz, 1H, H-2′′); 8.38 (dd, *J* = 7.5 and 0.9 Hz, 1H, H-8′′); 8.34 (m, 1H, H-4′); 8.07 (d, *J* = 8.2 Hz, 1H, H-4′′); 7.87 (d, *J* = 8.1 Hz, 1H, H-5′′); 7.80 (m, 1H, H-7′); 7.66–7.60 (m, 1H, H-3′′); 7.59–7.51 (m, 2H, H-6′′ and H-7′′); 7.33–7.27 (m, 2H, H-5′ and H-6′); 6.99–7.06 (m, 1H, H-5′′′); 6.98–6.94 (m, 2H, H-3′′′ and H-4′′′); 6.88 (d, *J* = 7.7 Hz, H-6′′′); 3.87 (s, OCH_3_); 3.73 (s, 2H, H-2); 3.15 (bs, 4H, H-3′′′′ and H-5′′′′); 2.82 (bs, 4H, H-2′′′′ and H-6′′′′). ^13^C-NMR δ (ppm): 193.8, 152.7, 141.5, 136.9, 134.9, 134.7, 133.8, 133.1, 130.9, 129.9, 129.7, 128.4, 128.2, 127.9, 126.0, 125.2, 124.6, 123.8, 123.6, 123.5, 121.4, 119.3, 118.8, 113.4, 111.7, 67.5, 55.8, 54.3(2C) and 51.0(2C). Elemental analysis for C_31_H_29_N_3_O_4_S (539.64 g/mol) calcd.: C: 69.00; H: 5.42; N: 7.79; S: 5.94. Found: C: 68.81; H: 5.34; N: 7.71; S: 6.06.

*1-(1-(Naphthalen-1-ylsulfonyl)-1H-indol-3-yl)-2-(4-(pyrimidin-2-yl)piperazin-1-yl)ethanone* (**3k**)


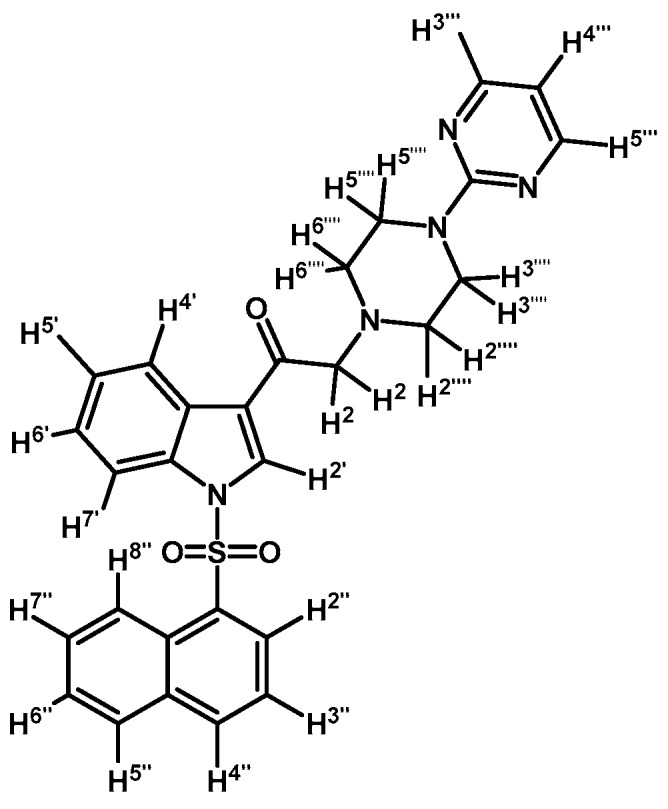


Prepared from 1-(2-pyrimidyl)-piperazine (96 mg, 0.583 mmol), potassium carbonate (80 mg, 0.583 mmol) and 2-bromo-1-(1-(naphthalen-1-ylsulfonyl)-1*H*-indol-3-yl)ethanone (**2e**) (250 mg, 0.583 mmol) to give a solid, which was purified by column chromatography on silica gel using CH_2_Cl_2_/acetone 9:1 to give 136 mg of (**3k**) as white crystalline plates. Yield: 46% m.p.: 88–89 °C; IR (KBr) cm^−1^: 1664, 1585, 1360, 1168, 1133. ^1^H-NMR δ (ppm): 8.98 (s, 1H, H-2′); 8.67 (d, *J* = 8.7 Hz, 1H, H-2′′); 8.38 (d, *J* = 7.4 Hz, 1H, H-8′′); 8.34 (dd, *J* = 3.2, 1H, H-4′); 8.32 (d, *J* = 4.8, 2H, H-4′′′ and H-6′′′); 8.04 (d, *J* = 8.2 Hz, 1H, H-4′′); 7.84 (d, *J* = 8.1 Hz, 1H, H-5′′); 7.79 (dd, *J* = 6.1 and 3.2 Hz, 1H, H-7′); 7.61 (t, *J* = 7.6 Hz, 1H, H-3′′); 7.57–7.47 (m, 2H, H-6′′ and H-7′′); 7.32–7.26 (m, 2H, H-5′ and H-6′); 6.49 (t, *J* = 4.7 Hz, 1H, H-5′′′); 3.87 (t, *J* = 4.9 Hz, 4H, H-3′′′′ and H-5′′′′); 3.69 (s, 2H, H-2); 2.64 (t, *J* = 4,9 Hz, 4H, H-2′′′′ and H-6′′′′). ^13^C-NMR δ (ppm): 193.6, 162.1, 158.1(2C), 136.9, 134.9, 134.7, 133.7, 133.1, 130.9, 129.9, 129.6, 128.4, 128.1, 127.9, 126.1, 125.2, 124.6, 123.7, 123.5, 119.3, 113.4, 110.5, 67.3, 53.8(2C) and 44.1(2C). Elemental analysis for C_28_H_25_N_5_O_3_S (511.59 g/mol) calcd.: C: 65.74; H: 4.93; N: 13.69; S: 6.27. Found: C: 65.61; H: 5.16; N: 13.52; S: 6.49.

*1-(1-(4-Methoxyphenylsulfonyl)-1H-indol-3-yl)-2-morpholinoethanone* (**3l**)


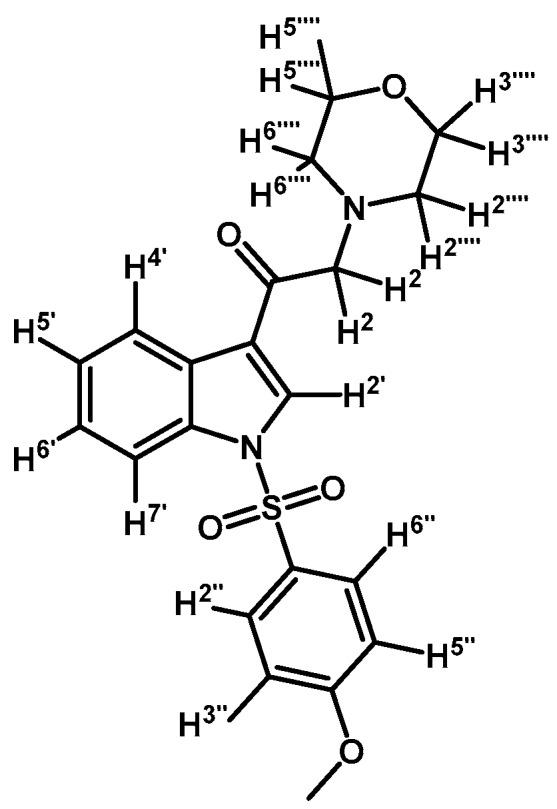


Prepared from morpholine (35 mg, 0.40 mmol), potassium carbonate (46 mg, 0.33 mmol) and 2-bromo-1-(1-(4-methoxyphenylsulfonyl)-1*H*-indol-3-yl)ethanone (**2f**) (136 mg, 0.33 mmol) to give a gel, which was purified by column chromatography on silica gel using CH_2_Cl_2_/acetone 9:1 to give 138 mg of product (**3l**) as a yellow gel. Yield: 81%; m.p.: product in gel state; IR (KBr) cm^−1^: 1665, 1378, 1169, 573. ^1^H-NMR δ (ppm): 8.66 (s, 1H, H-2′); 8.33 (dd, *J* = 6.5 and 1.3 Hz, 1H, H-4′); 7.93 (dd, *J* = 7.0 and 1.2 Hz, 1H, H-7′); 7.9 (d, *J* = 9.1 Hz, 2H, H-2′′ and H-6′′); 7.30–7.39 (m, 2H, H-5′ and H-6′); 6.92 (d, *J* = 9.1 Hz, 2H, H-3′′ and H-5′′); 3.76–3.79 (m, 7H, H-3′′′′, H-5′′′′ and OCH_3_); 3.68 (s, 2H, H-2); 2.61 (bs, 4H, H-2′′′′, H-6′′′′). ^13^C-NMR δ (ppm): 193.3, 164.8, 134.9, 133.2, 129.9 (2C), 129.2, 128.3, 126.1, 125.2, 123.4, 119.6, 115.3 (2C), 113.4, 67.3 (2C), 67.1, 56.2, 54.2 (2C). Elemental analysis for C_21_H_22_N_2_O_5_S (414.47 g/mol) calcd.: C: 60.85; H: 5.35; N: 6.76; S: 7.74. Found: C: 60.70; H: 5.53; N: 6.85; S: 7.80.

*1-(1-(3,5-Difluorophenylsulfonyl)-1H-indol-3-yl)-2-morpholinoethanone* (**3m**)


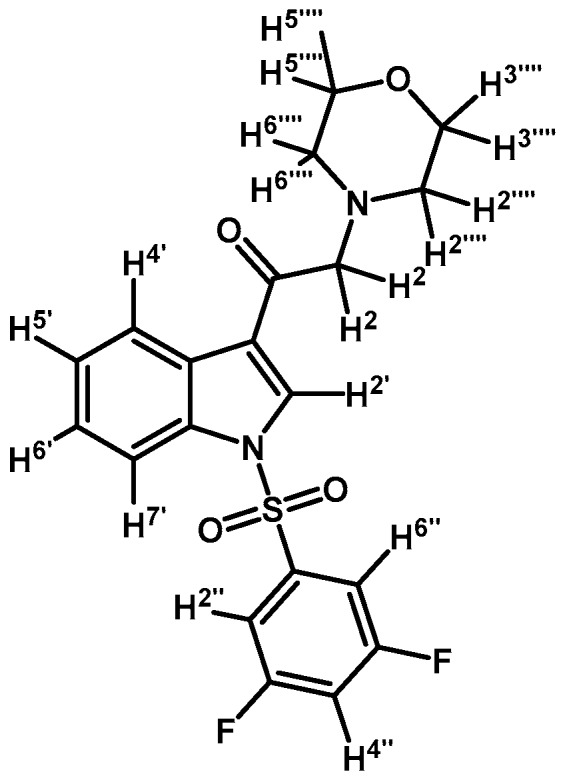


Prepared from morpholine (37 mg, 0.42 mmol), potassium carbonate (70 mg, 0.54 mmol) and 2-bromo-1-(1-(3,5-difluorophenylsulfonyl)-1*H*-indol-3-yl)ethanone (**2g**) (174 mg, 0.42 mmol) to give a gel, which was purified by column chromatography on silica gel using CH_2_Cl_2_/acetone 9:1 to give 142 mg of product (**3m**) as an orange gel. Yield: 80%; m.p.: product in gel state; IR (KBr) cm^−1^: 1685, 1607, 1444, 1300, 1389, 1173, 1132, 992, 614. ^1^H-NMR δ (ppm): 8.63 (s, 1H, H-2′); 8.35 (d, *J* = 7.4 Hz, 1H, H-4′); 7.92 (d, *J* = 7.9 Hz, 1H, H-7′); 7.49 (bs, 2H, H-2′′ and H-6′′); 7.40 (m, 2H, H-5′ and H-6′); 7.06 (t, *J* = 7.7 Hz, 1H, H-4′′); 3.78 (bs, 4H, H-3′′′ and H-5′′′); 3.67 (s, 1H, H-2); 2.61 (bs, 4H, H-2′′′ and H-6′′′). ^13^C-NMR δ (ppm): 193.3, 163.3 (dd, *J* = 256.9 and 11.7 Hz, 2C), 140.8 (t, *J* = 8.9 Hz, 1C), 134.8, 132.8, 128.4, 126.7, 125.8, 123.8, 120.7, 113.3, 111.1 (m, 3C), 67.5, 67.3 (2C), 54.3 (2C). Elemental analysis for C_20_H_18_F_2_N_2_O_4_S (420.43 g/mol) calcd.: C: 57.14; H: 4.32; N: 6.66; S: 7.63. Found: C: 57.27; H: 4.53; N: 6.81; S: 7.77.

#### 4.3.3. General Procedure for 2-(4-(Aryl)piperazin-1-yl)-1-(1-arylsulfonyl-1*H*-indol-3-yl)ethanol Derivatives (**4a**–**m**)

*2-(4-(Pyridin-2-yl)piperazin-1-yl)-1-(1-tosyl-1H-indol-3-yl)ethanol* (**4a**)


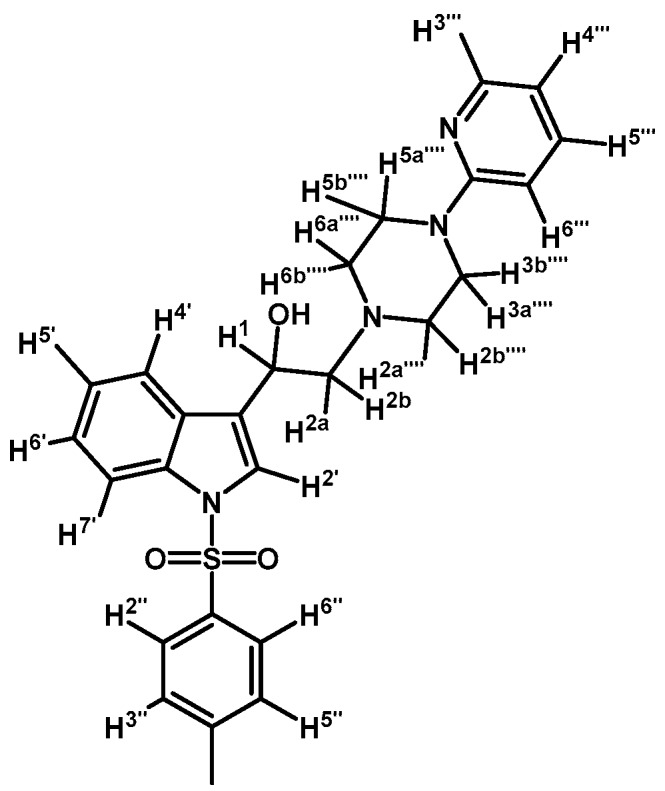


To a solution of (**3a**) (200 mg, 0.419 mmol) in ethanol (30 mL) was added sodium borohydride (17 mg, 0.450 mmol) in one portion and mixture was vigorously stirred at room temperature until the starting material had disappeared by checking TLC. Adding water (30 mL) stopped the reaction. The organic phase was extracted with CH_2_Cl_2_ (3 × 30 mL). The combined organic layers were dried with anhydrous sodium sulfate and removal of the solvent under vacuum afforded a residue, which was further purified by column chromatography on silica gel using CH_2_Cl_2_/acetone 9:1 to give 200 mg of (**4a**) as white crystalline plates. Yield: 98%; m.p.: 72.4–73.9 °C; IR (KBr) cm^−1^: 3422, 1595, 1438, 1369, 1174, 574. ^1^H-NMR δ (ppm): 8.13 (dd, *J* = 4.7 and 1.2 Hz, 1H, H-3′′′′); 7.91 (d, *J* = 8.3 Hz, 1H, H-4′); 7.69 (d, *J* = 8.3 Hz, 2H, H-2′′ and H-6′′); 7.55 (d, *J* = 7.8 Hz, 1H, H-7′); 7.51 (s, 1H, H-2′); 7.45–7.38 (m, 1H, H-5′′′′); 7.23 (t, *J* = 7.4 Hz, 1H, H-6′); 7.19–7.14 (m, 1H, H-5′); 7.13 (d, *J* = 8.1 Hz, 2H, H-3′′ and H-5′′); 6.61–6.53 (m, 2H, H-4′′′′ and H-6′′′′); 4.98 (dd, *J* = 10.1 and 3.1 Hz, 1H, H-1); 3.59–3.44 (m, 4H, H-3′′′′ and H-5′′′′); 2.82–2.75 (m, 2H, H-2a′′′′ and H-6a′′′′); 2.70 (d, *J* = 10.3 Hz, 1H, H-2a); 2.63 (dd, *J* = 12.5 and 3.4 Hz, 1H, H-2b); 2.55–2.48 (m, 2H, H-2b′′′′ and H-6b′′′′); 2.25 (s, 3H, CH_3_). ^13^C-NMR δ (ppm): 159.4, 148.0, 145.0, 137.6, 135.5, 135.3, 129.9 (2C), 128.9, 126.9 (2C), 124.8, 123.2, 123.1, 123.0, 120.3, 113.8, 113.6, 107.2, 64.0, 63.2, 53.0 (2C), 45.4 (2C) and 22.7. Elemental analysis for C_26_H_28_N_4_O_3_S (476.59 g/mol) calcd.: C: 65.52; H: 5.92; N: 11.76; S: 6.73. Found: C: 65.37; H: 5.83; N: 11.50; S: 6.79.

*2-(4-(2-Methoxyphenyl)piperazin-1-yl)-1-(1-tosyl-1H-indol-3-yl)ethanol* (**4b**)


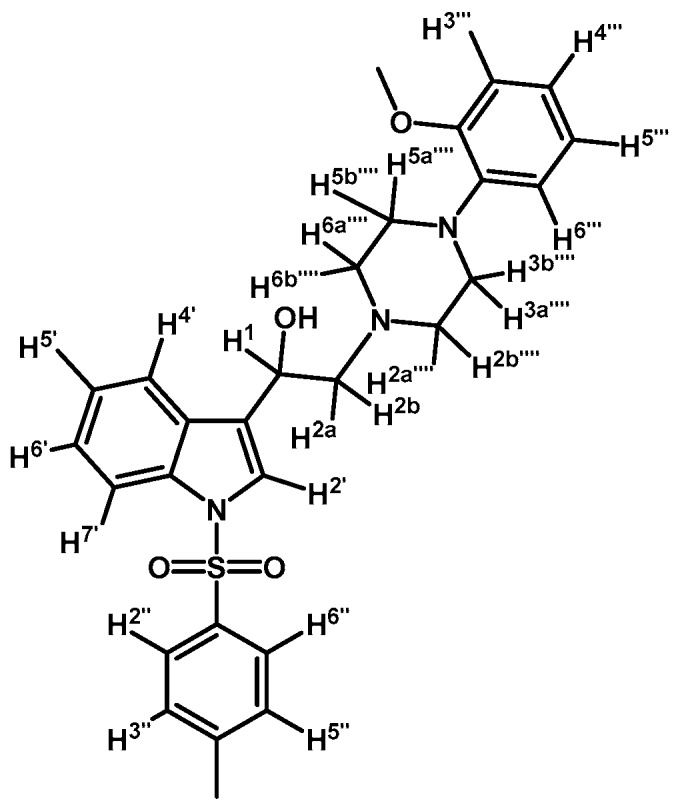


Prepared from (**3b**) (200 mg, 0.395 mmol) and sodium borohydride (17 mg, 0.450 mmol) to give a solid, which was purified by column chromatography on silica gel using AcOEt/hexane 9:1 to obtain 197 mg of product (**4b**) as white crystalline plates. Yield: 99%; m.p.: 86.3–86.9 °C; IR (KBr) cm^−1^: 3446, 1371, 1174, 1241, 1121, 748, 574. ^1^H-NMR δ (ppm): 8.00 (d, *J* = 8.3 Hz, 1H, H-4′), 7.80 (d, *J* = 8.2 Hz, 2H, H-2′′, H-6′′), 7.65 (d, *J* = 7.8 Hz, 1H, H-7′), 7.60 (s, 1H, H-2′), 7.34 (t, *J* = 7.6 Hz, 1H, H-6′), 7.22–7.27 (m, 3H, H-5′, H-3′′ and H-5′′), 7.08–7.01 (m, 1H, H-5′′′′), 7.01–6.93 (m, 2H, H-3′′′′ and H-4′′′′), 6.90 (d, *J* = 7.9 Hz, 1H, H-6′′′′), 5.06 (dd, *J* = 9.6, 3.9 Hz, 1H, H-1), 3.90 (s, 3H, OCH_3_), 3.16 (bs, 4H, H-3′′′ and H-5′′′), 2.99 (bs, 2H, H-2′′′), 2.85–2.75 (m, 2H, H-2), 2.71 (bs, 2H, H-6′′′), 2.36 (s, 3H, CH_3_), 1.70 (bs, 1H, OH). ^13^C-NMR δ (ppm): 152.6, 145.4, 141.4, 135.9, 135.6, 130.3 (2C), 129.4, 127.3 (2C), 125.2, 123.6, 123.5, 123.5, 123.4, 121.4, 120.7, 118.6, 114.2, 111.6, 64.3, 63.4, 55.8, 53.8, 51.2, 31.4 (2C) and 22.0. Elemental analysis for C_28_H_31_N_3_O_4_S (505.63 g/mol) Calcd.: C: 66.51; H: 6.18; N: 8.31; S: 6.34. Found: C: 66.21; H: 6.00; N: 8.09; S: 6.63.

*2-(4-(Pyrimidin-2-yl)piperazin-1-yl)-1-(1-tosyl-1H-indol-3-yl)ethanol* (**4c**)


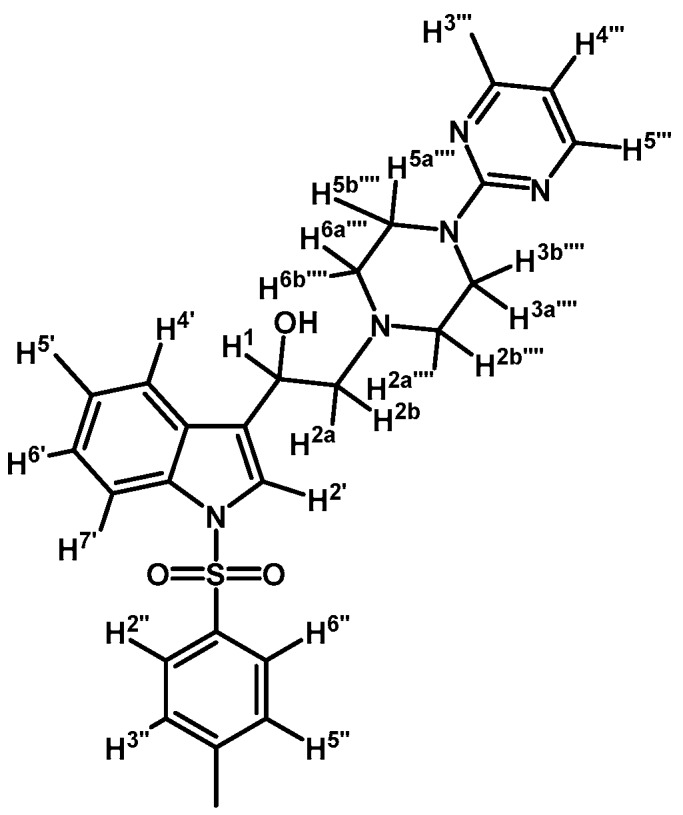


Prepared from (**3c**) (75 mg, 0.157 mmol) and sodium borohydride (10 mg, 0.264 mmol) to give a solid, which was purified by column chromatography on silica gel using AcOEt/hexane 9:1 to obtain 51 mg of (**4c**) as white crystalline plates. Yield: 68%; m.p.: 81–82 °C; IR (KBr) cm^−1^: 3423, 1586, 1360, 1174, 574. ^1^H-NMR δ (ppm): 8.32 (d, *J* = 4.6 Hz, 2H, H-4′′′ and H-6′′′); 7.98 (d, *J* = 8.3 Hz, 1H, H-4′); 7.77 (d, *J* = 8.0 Hz, 2H, H-2′′ and H-6′′); 7.62 (d, *J* = 7.8 Hz, 1H, H-7′); 7.58 (s, 1H, H-2′); 7.31 (t, *J* = 7.7 Hz, 1H, H-6′); 7.27–7.18 (m, 3H, H-5′, H-3′′ and H-5′′); 6.51 (t, *J* = 4.6 Hz, 1H, H-5′′′); 5.1 (dd, *J* = 10.2 and 2.8 Hz, 1H, H-1); 3.82–3.95 (m, 4H, H-3′′′′ and H-5′′′′); 2.75–2.86 (m, 3H, H-2a′′′′, H-6a′′′′ and H-2a); 2.70 (dd, *J* = 12.5 and 3.0 Hz, 1H, H-2b); 2.50–2.59 (m, 2H, H-2b′′′′ and H-6b′′′′); 2.34 (s, 3H, CH_3_). ^13^C-NMR δ (ppm): 162.0, 158.2 (2C), 145.4, 135.9, 135.7, 130.3 (2C), 129.4, 127.3 (2C), 125.2, 123.5 (2C), 123.4, 120.7, 114.2, 110.5, 64.5, 63.6, 53.5 (2C), 44.2 (2C) and 22.0. Elemental analysis for C_25_H_27_N_5_O_3_S (477.58 g/mol) calcd.: C: 62.87; H: 5.70; N: 14.66; S: 6.71. Found: C: 62.67; H: 5.93; N: 14.82; S: 6.76.

*1-(1-(4-Chlorophenylsulfonyl)-1H-indol-3-yl)-2-(4-(pyrimidin-2-yl)piperazin-1-yl)ethanol* (**4d**)


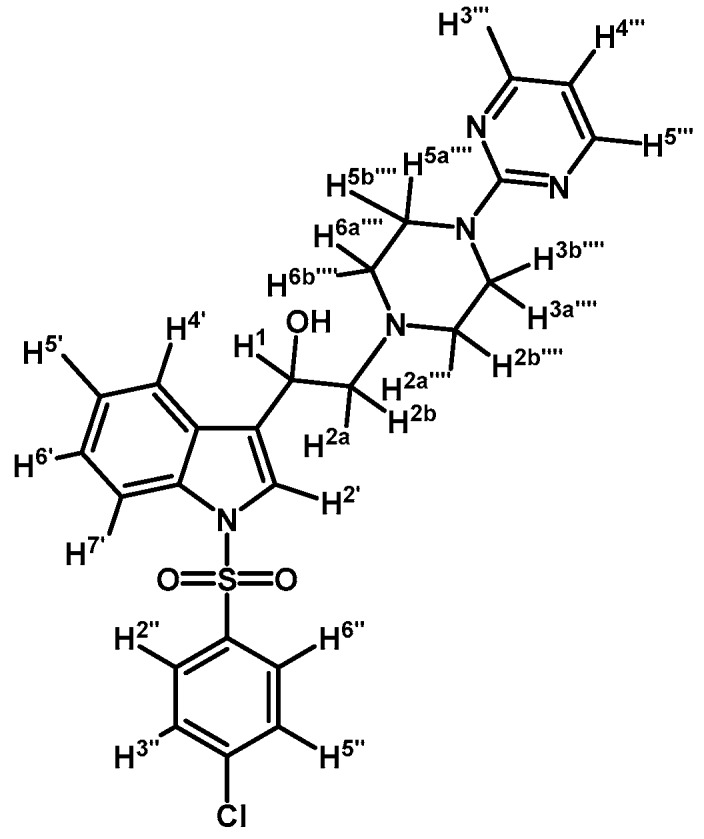


Prepared from (**3d**) (170 mg, 0.343 mmol) and sodium borohydride (16 mg, 0.422 mmol) to give a solid, which was purified by column chromatography on silica gel using AcOEt/hexane 9:1 to yield 126 mg of compound (**4d**) as pale brown crystalline plates. Yield: 75%; m.p.: 81–82 °C; IR (KBr) cm^−1^: 3423, 1586, 1360, 1176, 570. ^1^H-NMR δ (ppm): 8.32 (d, *J* = 4.7 Hz, 2H, H-4′′′ and H-6′′′); 7.96 (d, *J* = 8.3 Hz, 1H, H-4′); 7.81 (d, *J* = 8.6 Hz, 2H, H-2′′ and H-6′′); 7.62 (d, *J* = 7.8 Hz, 1H, H-7′); 7.56 (s, 1H, H-2′); 7.39 (d, *J* = 8.6 Hz, 2H, H-3′′ and H-5′′); 7.33 (t, *J* = 7.7, 1H, H-6′); 7.25 (t, *J* = 7.5 Hz, 1H, H-5′); 6.51 (t, *J* = 4.7 Hz, 1H, H-5′′′); 5.08 (dd, *J* = 10.0 and 3.2 Hz, 1H, H-1); 3.83-3.92 (m, 4H, H-3′′′′ and H-5′′′′); 3.37 (bs, 1H, OH); 2.79–2.87 (m, 2H, H-2a′′′′ and H-6a′′′′); 2.77 (d, *J* = 10.3 Hz, 1H, H-2a); 2.72 (dd, *J* = 12.5 and 3.4 Hz, 1H, H-2b); 2.52–2.61 (m, 2H, H-2b′′′′ and H-6b′′′′). ^13^C-NMR δ (ppm): 162.0, 158.2 (2C), 141.0, 136.9, 135.8, 130.1 (2C), 129.4, 128.6 (2C), 125.5, 124.3, 123.9, 123.2, 120.9, 114.1, 110.6, 64.5, 63.5, 53.5 (2C), 44.1 (2C). Elemental analysis for C_24_H_24_ClN_5_O_3_S (498.00 g/mol) calcd.: C: 57.88; H: 4.86; N: 14.06; S: 6.44. Found: C: 57.97; H: 4.88; N: 13.69; S: 6.78.

*1-(1-(4-Fluorophenylsulfonyl)-1H-indol-3-yl)-2-(4-(pyrimidin-2-yl)piperazin-1-yl)ethanol* (**4e**)


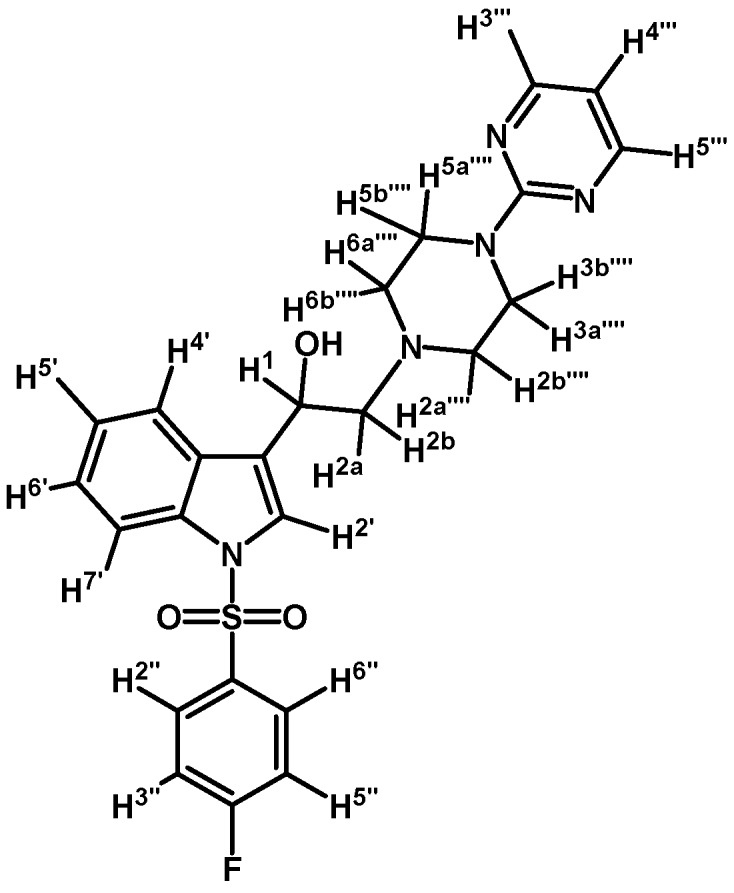


Prepared from (**3e**) (140 mg, 0.292 mmol) and sodium borohydride (13 mg, 0.340 mmol) to give a solid, which was purified by column chromatography on silica gel using AcOEt/hexane 9:1 to obtain 97 mg of compound (**4e**) as pale yellow crystalline plates. Yield: 69%; m.p.: 72–73 °C; IR (KBr) cm^−1^: 3422, 1587, 1360, 1180, 982, 573. ^1^H-NMR δ (ppm): 8.32 (d, *J* = 4.7 Hz, 2H, H-4′′′ and H-6′′′); 7.97 (d, *J* = 8.3 Hz, 1H, H-4′); 7.90 (dd, *J* = 8.9 and 4.9, 2H, H-2′′ and H-6′′); 7.63 (d, *J* = 7.8 Hz, 1H, H-7′); 7.57 (s, 1H, H-2′); 7.19–7.35 (m, 2H, H-5′ and H-6′); 7.09 (t, *J* = 8.5 Hz, 2H, H-3′′ and H-5′′); 6.50 (t, *J* = 4.7 Hz, 1H, H-5′′′); 5.07 (dd, *J* = 10.2 and 3.3 Hz, 1H, H-1); 3.84–3.95 (m, 4H, H-3′′′′ and H-5′′′′); 3.46 (bs, 1H, OH); 2.77–2.86 (m, 2H, H-2a′′′′ and H-6a′′′′); 2.77 (d, *J* = 10.2 Hz, 1H, H-2a); 2.71 (dd, *J* = 12.6 and 3.5 Hz, 1H, H-2b); 2.52–2.59 (m, 2H, H-2b′′′′ and H-6b′′′′). ^13^C-NMR δ (ppm): 166.1 (d, *J* = 257.2 Hz, 1C); 162.0; 158.2 (2C); 135.8; 134.6 (d, *J* = 3.1 Hz, 1C); 130.1 (d, *J* = 9.7 Hz, 2C); 125.4; 124.2; 123.8; 123.3; 120.8; 117.1 (d, *J* = 22.9 Hz, 2C); 115.2; 114.1; 110.5; 64.5; 63.6; 53.5 (2C); 44.2 (2C). Elemental analysis for C_24_H_24_FN_5_O_3_S (481.54 g/mol) calcd.: C: 59.86; H: 5.02; N: 14.54; S: 6.66. Found: C: 59.82; H: 5.13; N: 14.29; S: 6.99.

*1-(1-(4-Iodophenylsulfonyl)-1H-indol-3-yl)-2-(4-(pyridin-2-yl)piperazin-1-yl)ethanol* (**4f**)


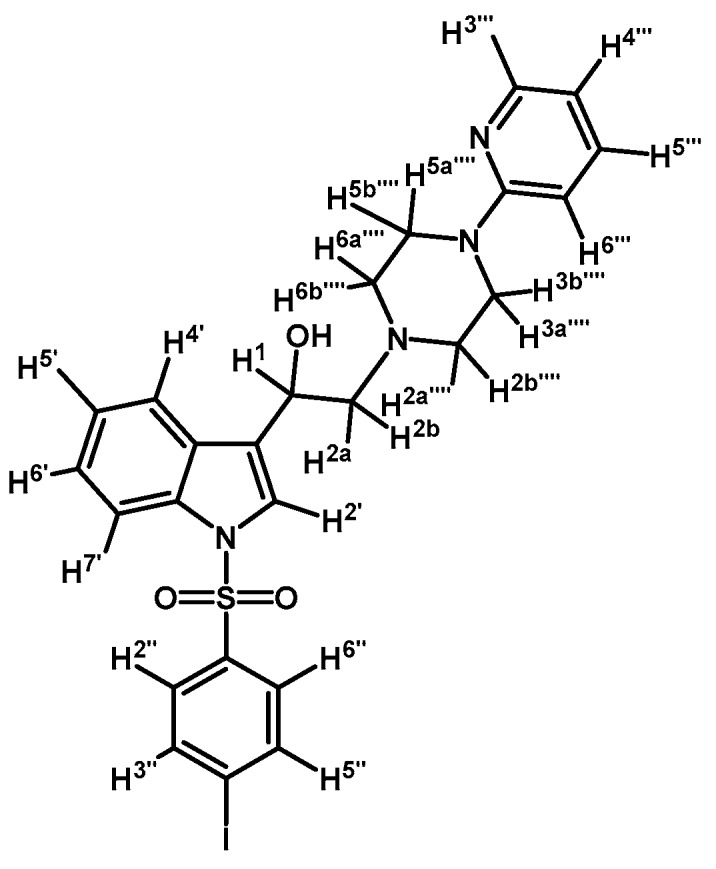


Prepared from (**3f**) (130 mg, 0.222 mmol) and sodium borohydride (10 mg, 0.269 mmol) to give a solid, which was purified by column chromatography on silica gel using AcOEt/hexane 9:1 to obtain 72 mg of compound (**4f**) as pale brown crystalline plates. Yield: 56%; m.p.: 157–158 °C; IR (KBr) cm^−1^: 3382, 1592, 1384, 1174, 1122, 1098, 607, 568. ^1^H-NMR δ (ppm): 8.20 (dd, *J* = 4.8 and 1.2 Hz, 1H, H-3′′′); 7.96 (d, *J* = 8.3 Hz, 1H, H-4′); 7.76 (d, *J* = 8.6 Hz, 2H, H-2′′ and H-6′′); 7.62 (d, *J* = 7.8 Hz, 1H, H-7′); 7.57 (d, *J* = 8.7 Hz, 2H, H-3′′ and H-5′′); 7.55 (s, 1H, H-2′); 7.45–7.53 (m, 1H, H-5′′′); 7.33 (t, *J* = 7.3 Hz, 1H, H-5′); 7.25 (t, *J* = 7.2 Hz, 1H, H-6′); 6.62–6.68 (m, 2H, H-4′′′ and H-6′′′), 5.06 (dd, *J* = 9.6 and 3.7 Hz, 1H, H-1); 3.52–3.66 (m, 4H, H-3′′′′ and H-5′′′′); 2.81–2.91 (m, 2H, H-2a′′′′ and H-6a′′′′); 2.69–2.80 (m, 2H, H-2a and H-2b); 2.56–2.63 (m, 2H, H-2b′′′′ and H-6b′′′′). ^13^C-NMR δ (ppm): 159.8; 148.4; 139.0 (2C); 138.1; 138.0; 135.8; 129.4; 128.4 (2C); 125.5; 124.4; 123.9; 123.2; 120.9; 114.1; 114.0; 107.6; 102.2; 64.4; 63.6; 53.4 (2C); 45.8 (2C). Elemental analysis for C_25_H_25_IN_4_O_3_S (588.46 g/mol) calcd.: C: 51.03; H: 4.28; N: 9.52; S: 5.45. Found: C: 50.85; H: 4.44; N: 9.43; S: 5.51.

*1-(1-(4-Iodophenylsulfonyl)-1H-indol-3-yl)-2-(4-(2-methoxyphenyl)piperazin-1-yl)ethanol* (**4g**)


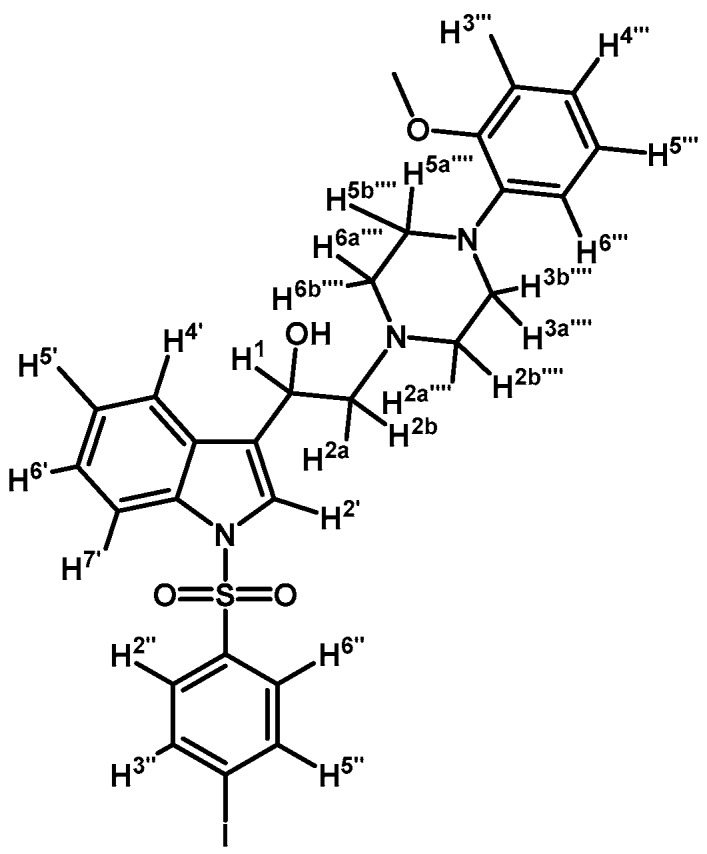


Prepared from (**3g**) (138 mg, 0.224 mmol) and sodium borohydride (10 mg, 0.269 mmol) to give a solid, which was purified by column chromatography on silica gel using AcOEt/hexane 9:1 to obtain 86 mg of compound (**4g**) as pale brown crystalline plates. Yield: 62%; m.p.: 109–110 °C; IR (KBr) cm^−1^: 3423, 1500, 1447, 1385, 1175, 1241, 1120, 748, 734, 608, 568. ^1^H-NMR δ (ppm): 7.96 (d, *J* = 8.2 Hz, 1H, H-4′); 7.75 (d, *J* = 8.4 Hz, 2H, H-2′′ and H-6′′); 7.63 (d, *J* = 7.8 Hz, 1H, H-7′); 7.56 (d, *J* = 8.3 Hz, 3H, H-2′, H-3′′ and H-5′′); 7.33 (t, *J* = 7.7 Hz, 1H, H-6′); 7.25 (t, *J =* 7.4 Hz, 1H, H-5′); 6.98-7.06 (m, 1H, H-5′′′); 6.90–6.97 (m, 2H, H-3′′′ and H-4′′′); 6.87 (d, *J* = 7.9 Hz, 1H, H-6′′′); 5.05 (t, *J* = 6.7 Hz, 1H, H-1); 3.87 (s, 3H, OCH_3_); 3.14 (bs, 4H, H-3′′′′ and H-5′′′′); 2.97 (bs, 2H, H-2a′′′′ and H-6a′′′′); 2.77 (d, *J* = 6.5 Hz, 2H, H-2); 2.70 (bs, 2H, H-2b′′′′ and H-6b′′′′). ^13^C-NMR δ (ppm): 152.7; 141.5; 139.0 (2C); 138.1; 135.8; 129.5; 128.4 (2C); 125.5; 124.6; 123.9; 123.6; 123.2; 121.5; 120.9; 118.7; 114.1; 111.7; 102.2; 64.4; 63.5; 55.8 (2C); 53.8; 51.1 (2C). Elemental analysis for C_27_H_28_IN_3_O_4_S (617.50 g/mol) calcd.: C: 52.52; H: 4.57; N: 6.80; S: 5.19 Found: C: 52.40; H: 4.75; N: 6.65; S: 5.55.

*1-(1-(4-Iodophenylsulfonyl)-1H-indol-3-yl)-2-(4-(pyrimidin-2-yl)piperazin-1-yl)ethanol* (**4h**)


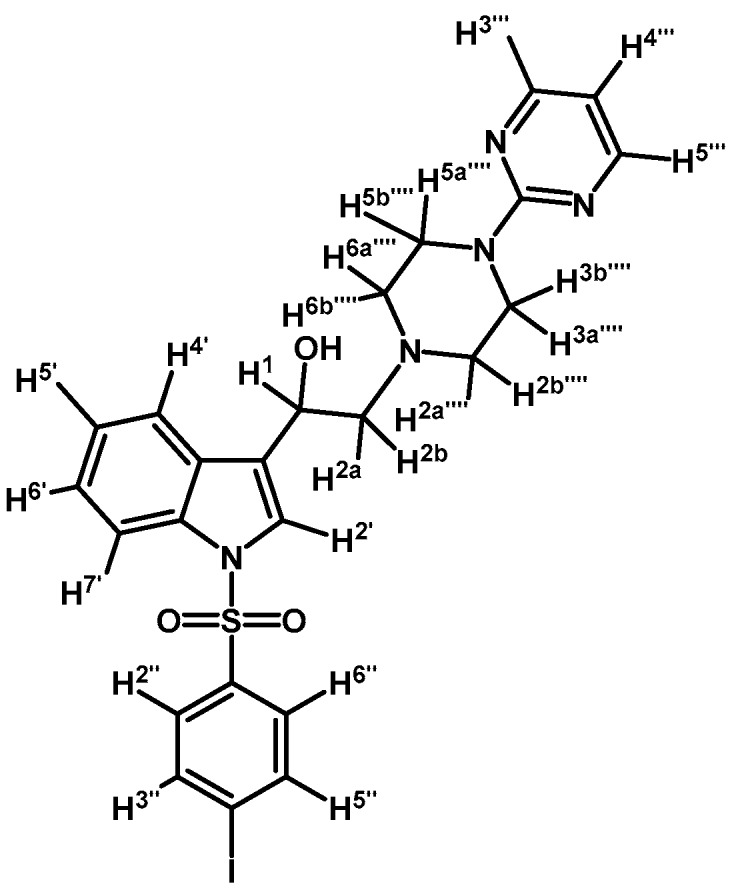


Prepared from (**3h**) (147 mg, 0.25 mmol) and sodium borohydride (12 mg, 0.3 mmol) to give a solid, which was purified by column chromatography on silica gel using AcOEt/hexane 9:1 to yield 98 mg of (**4h**) as brown crystalline plates. Yield: 67%; m.p.: 171–172 °C; IR (KBr) cm^−1^: 3451, 1585, 1485, 1449, 1359, 1174, 1125, 982, 609, 569. ^1^H-NMR δ (ppm): 8.31 (d, *J* = 4.8 Hz, 2H, H-4′′′ and H-6′′′); 7.96 (d, *J* = 8.3 Hz, 1H, H-4′); 7.74 (d, *J* = 8.6 Hz, 2H, H-2′′ and H-6′′); 7.62 (d, *J* = 7.8 Hz, 1H, H-7′); 7.56 (d, *J* = 8.5 Hz, 2H, H-3′′ and H-5′′); 7.55 (s, 1H, H-2′); 7.32 (t, *J* = 7.4 Hz, 1H, H-6′); 7.24 (t, *J* = 7.3 Hz, 1H, H-5′); 6.50 (t, *J* = 4.7, 1H, H-5′′′); 5.06 (dd, *J* = 10.0 and 3.3 Hz, 1H, H-1); 3.82–3.94 (m, 4H, H-3′′′′ and H-5′′′′); 2.76–2.84 (m, 2H, H-2a′′′′ and H-6a′′′′); 2.75 (d, *J* = 10.1 Hz, 1H, H-2a); 2.70 (dd, *J* = 12.6 and 3.7 Hz, 1H, H-2b); 2.51–2.57 (m, 2H, H-2b′′′′ and H-6b′′′′). ^13^C-NMR δ (ppm): 162.0; 158.2 (2C); 139.0 (2C); 138.1; 135.8; 129.4; 128.4 (2C); 125.5; 124.4; 123.9; 123.2; 120.9; 114.1; 110.5; 102.2; 64.5; 63.6; 53.5 (2C); 44.2 (2C). Elemental analysis for C_24_H_24_IN_5_O_3_S (589.45 g/mol) calcd.: C: 48.90; H: 4.10; N: 11.88; S: 5.44. Found: C: 48.71; H: 4.28; N: 11.67; S: 5.58.

*1-(1-(Naphthalen-1-ylsulfonyl)-1H-indol-3-yl)-2-(4-(pyridin-2-yl)piperazin-1-yl)ethanol* (**4i**)


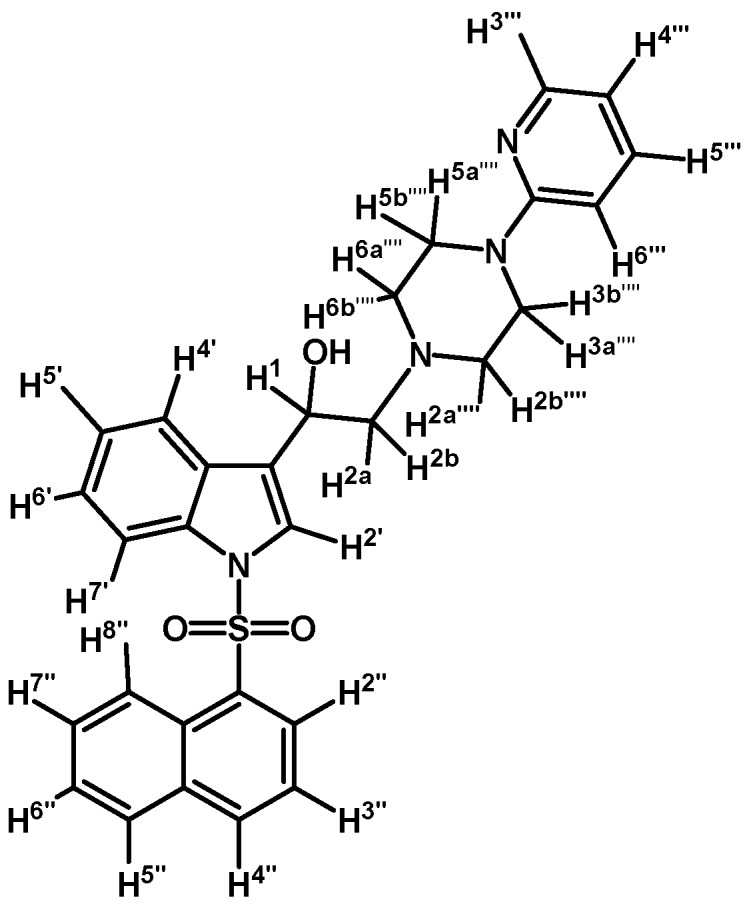


Prepared from (**3i**) (110 mg, 0.216 mmol) and sodium borohydride (10 mg, 0.269 mmol) to give a solid, which was purified by column chromatography on silica gel using AcOEt/hexane 9:1 to obtain 80 mg of compound (**4i**) as pale brown crystalline plates. Yield: 72%; m.p.: 80–81°C; IR (KBr) cm^−1^: 3423, 1594, 1437, 1361, 1171, 1122, 981, 769, 600. ^1^H-NMR δ (ppm): 8.72 (d, *J* = 8.6 Hz, 1H, H-2′′); 8.20 (d, *J* = 3.8 Hz, 1H, H-3′′′); 8.10 (d, *J* = 7.4 Hz, 1H, H-4′′); 8.02 (d, *J* = 8.2 Hz, 1H, H-4′); 7.85 (d, *J* = 9.3 Hz, 1H, H-7′); 7.83 (d, *J* = 9.1 Hz, 1H, H-8′′); 7.78 (s, 1H, H-2′); 7.60–7.65 (m, 2H, H-3′′ and H-5′′); 7.53 (t, *J* = 7.6 Hz, 1H, H-6′); 7.44–7.50 (m, 2H, H-5′ and H-5′′′); 7.16–7.27 (m, 2H, H-6′′ and H-7′′); 6.61–6.67 (m, 2H, H-4′′′ and H-6′′′); 5.08 (dd, *J* = 10.1 and 3.0 Hz, 1H, H-1); 3.51–3.64 (m, 4H, H-3′′′′ and H-5′′′′); 2.80–2.88 (m, 2H, H-2a′′′′ and H-6a′′′′); 2.77 (d, *J* = 10.3 Hz, 1H, H-2a); 2.69 (dd, *J* = 12.5 and 3.4 Hz, H-2b); 2.55–2.63 (m, 2H, H-2b′′′′ and H-6b′′′′). ^13^C-NMR δ (ppm): 159.8; 148.4; 137.9; 135.9; 135.8; 134.7; 134.5; 129.6; 129.5; 129.2; 129.0; 128.6; 127.6; 125.2; 124.5; 124.4; 123.9; 123.5; 122.9; 120.8; 114.0; 113.9; 107.6; 64.5; 63.7; 53.4 (2C); 45.8 (2C).Elemental analysis for C_29_H_28_N_4_O_3_S (512.62 g/mol) calcd.: C: 67.95; H: 5.51; N: 10.93; S: 6.26 Found: C: 68.10; H: 5.73; N: 11.16; S: 6.02.

*2-(4-(2-Methoxyphenyl)piperazin-1-yl)-1-(1-(naphthalen-1-ylsulfonyl)-1H-indol-3-yl)ethanol* (**4j**)


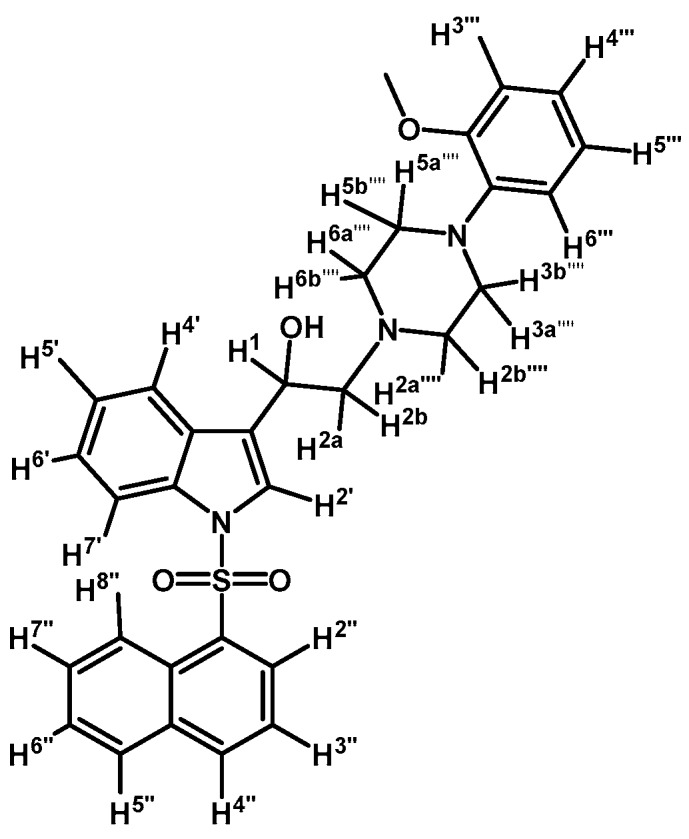


Prepared from (**3j**) (82 mg, 0.15 mmol) and sodium borohydride (7 mg, 0.19 mmol) to give a solid, which was purified by column chromatography on silica gel using AcOEt/hexane 9:1 to obtain 80 mg of compound (**4j**) as light brown crystalline plates. Yield: 61%; m.p.: 89–90 °C; IR (KBr) cm^−1^: 3423, 1500, 1448, 1361, 1172, 1241, 1121, 747. ^1^H-NMR δ (ppm): 8.73 (d, *J* = 8.6 Hz, 1H, H-2′′); 8.08 (d, *J* = 7.4 Hz, 1H, H-4′′); 8.04 (d, *J* = 8.2 Hz, 1H, H-4′); 7.87 (d, *J* = 8.1 Hz, 1H, H-7′); 7.83 (d, *J* = 8.2 Hz, 1H, H-8′′); 7.78 (s, 1H, H-2′); 7.61–7.67 (m, 2H, H-3′′ and H-5′′); 7.55 (t, *J* = 7.5 Hz, 1H, H-6′); 7.49 (t, *J* = 7.9 Hz, 1H, H-5′); 7.18–7.28 (m, 2H, H-6′′ and H-7′′); 7.00–7.06 (m, 1H, H-5′′′); 6.92–6.96 (m, 2H, H-3′′′ and H-4′′′); 6.88 (d, *J* = 7.9 Hz, 1H, H-6′′′); 5.1 (dd, *J* = 9.7 and 3.2 Hz, 1H, H-1); 3.87 (s, 3H, OCH_3_); 3.15 (bs, 4H, H-3′′′′ and H-5′′′′); 2.98 (bs, 2H, H-2a′′′′ and H-6a′′′′); 2.71–2.85 (m, 4H, H-2a, H-2b, H-2b′′′′ and H-6b′′′′). ^13^C-NMR δ (ppm): 152.7; 141.5; 135.9; 135.8; 134.7; 134.5; 129.5 (2C); 129.1; 129.0; 128.6; 127.6; 125.1 (2C); 124.5; 123.9; 123.6; 123.5; 123.0; 121.5; 120.8; 118.7; 114.0; 111.7; 64.4; 63.5; 55.8 (2C); 53.8; 51.1 (2C). Elemental analysis for C_31_H_31_N_3_O_4_S (541.66 g/mol) calcd.: C: 68.74; H: 5.77; N: 7.76; S: 5.92 Found: C: 68.58; H: 5.92; N: 7.62; S: 5.72.

*1-(1-(Naphthalen-1-ylsulfonyl)-1H-indol-3-yl)-2-(4-(pyrimidin-2-yl)piperazin-1-yl)ethanol* (**4k**)


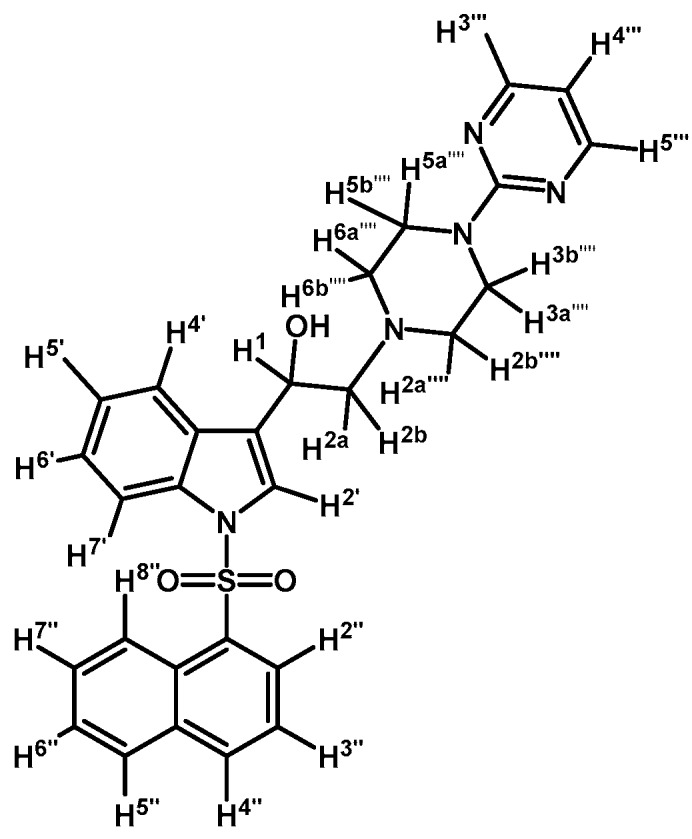


Prepared from (**3k**) (73 mg, 0,143 mmol) and sodium borohydride (10 mg, 0.26 mmol) to give a solid, which was purified by column chromatography on silica gel using AcOEt/hexane 9:1 to obtain 45 mg of compound (**4k**) as pale brown crystalline plates. Yield: 62%; m.p.: 82–83 °C; IR (KBr) cm^−1^: 3424, 1586, 1447, 1360, 1171, 1122, 983, 599. ^1^H-NMR δ (ppm): 8.72 (d, *J* = 8.7 Hz, 1H, H-2′′); 8.31 (d, *J* = 4.7 Hz, 2H, H-4′′′ and H-6′′′); 8.09 (d, *J* = 7.4 Hz, 1H, H-4′′); 8.02 (d, *J* = 8.2 Hz, 1H, H-4′); 7.85 (d, *J* = 8.2 Hz, 1H, H-7′); 7.82 (d, *J* = 8.4 Hz, 1H, H-8′′); 7.78 (s, 1H, H-2′); 7.60–7.65 (m, 2H, H-3′′ and H-5′′); 7.54 (t, *J* = 7.5 Hz, 1H, H-6′); 7.47 (t, *J* = 7.9 Hz, 1H, H-5′); 7.17–7.27 (m, 2H, H-6′′ and H-7′′), 6.50 (t, *J* = 4.7 Hz, 1H, H-5′′′), 5.09 (dd, *J* = 10.2 and 2.8 Hz, 1H, H-1); 3.83–3.93 (m, 4H, H-3′′′′ and H-5′′′′); 2.75 (m, 3H, H-2a′′′′, H-6a′′′′ and H-2a); 2.69 (dd, *J* = 12.5 and 3.1 Hz, H-1, H-2b); 2.51–2.58 (m, 2H, H-2b′′′′, H-6b′′′′). ^13^C-NMR δ (ppm): 162.0; 158.1 (2C), 135.9; 135.8; 134.7; 134.5; 129.6; 129.5; 129.1; 129.0; 128.6; 127.6; 125.2; 124.5; 124.4; 123.9; 123.5; 122.8; 120.8; 114.0; 110.5; 64.6; 63.6; 53.5 (2C); 44.2 (2C). Elemental analysis for C_28_H_27_N_5_O_3_S (513.61 g/mol) calcd.: C: 65.48; H: 5.30; N: 13.64; S: 6.24 Found: C: 65.57; H: 5.11; N: 13.49; S: 6.03.

*1-(1-(4-Methoxyphenylsulfonyl)-1H-indol-3-yl)-2-morpholinoethanol* (**4l**)


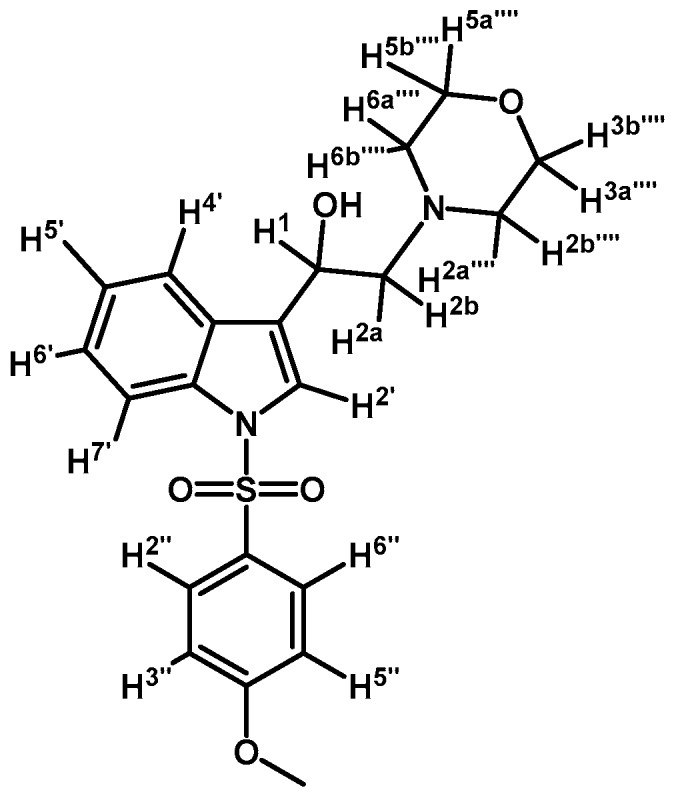


Prepared from (**3l**) (73 mg, 0.18 mmol) and sodium borohydride (67 mg, 1.77 mmol) to give a solid, which was purified by column chromatography on silica gel using AcOEt to yield 39 mg of compound (**4l**) as yellow crystalline plates. Yield: 53%; m.p.: 149–152 °C; IR (KBr) cm^−1^: 3454, 1595, 1364, 1270, 1166, 1117, 573. ^1^H-NMR δ (ppm): 7.97 (d, *J* = 8.3 Hz, 1H, H-4′); 7.81 (d, *J* = 9.0 Hz, 2H, H-2′′ and H-6′′); 7.60 (d, *J* = 7.8 Hz, 1H, H-7′); 7.57 (s, 1H, H-2′); 7.31 (t, *J* = 7.5 Hz, 1H, H-6′); 7.22 (t, *J* = 7.5 Hz, 1H, H-5′); 6.85 (d, *J* = 9.0 Hz, 2H, H-3′′ and H-5′′); 5.02 (dd, *J* = 9.1 and 4.4 Hz, 1H, H-1); 3.69–3.80 (m, 7H, H-3′′′′, H-5′′′′ and OCH_3_); 2.68–2.77 (m, 4H, H-2a′′′′, H-6a′′′′, H-2a and H-2b); 2.45–2.51 (m, 2H, H-2b′′′′ and H-6b′′′′). ^13^C-NMR δ (ppm): 164.2, 135.8, 130.1, 129.5 (2C), 129.3, 125.2, 123.5, 123.4 (2C), 120.6, 114.9 (2C), 114.2, 67.4 (2C), 64.9, 63.4, 56.1 (2C), 54.0. Elemental analysis for C_21_H_24_N_2_O_5_S (416.49 g/mol) calcd.: C: 60.56; H: 5.81; N: 6.73; S: 7.70 Found: C: 60.42; H: 5.88; N: 6.79; S: 7.57.

*1-(1-(3,5-Difluorophenylsulfonyl)-1H-indol-3-yl)-2-morpholinoethanol* (**4m**)


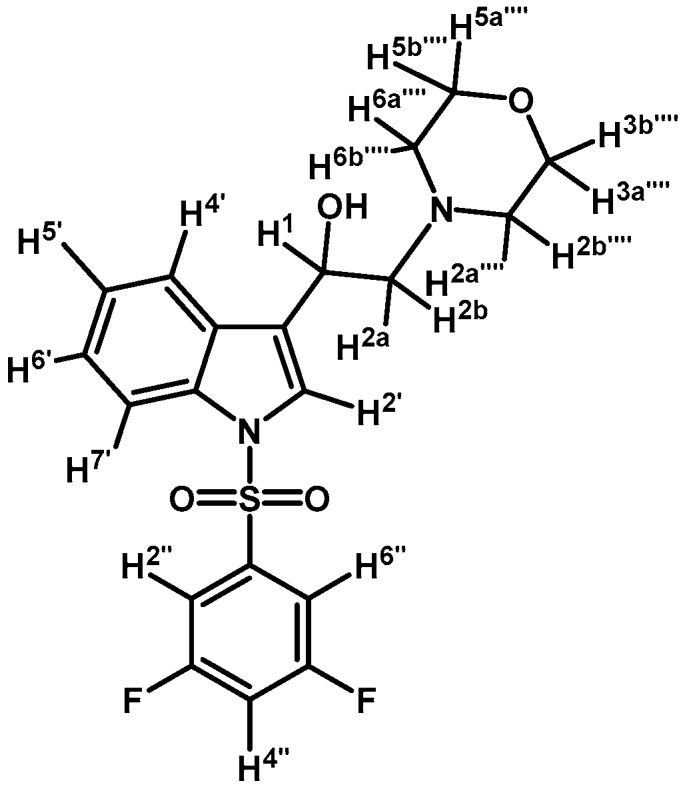


Prepared from (**3m**) (70 mg, 0.167 mmol) and sodium borohydride (10 mg, 0.26 mmol) to give an oil, which was purified by column chromatography on silica gel using AcOEt to obtain 46 mg of (**4m**) as a yellow oil. Yield: 66%; m.p.: product is an oil; IR (KBr) cm^−1^: 3381 (OH), 1606, 1444, 1384 and 1179, 1298, 1115, 617. ^1^H-NMR δ (ppm): 7.96 (d, *J* = 8.3 Hz, 1H, H-4′); 7.63 (d, *J* = 7.8 Hz, 1H, H-7′); 7.52 (d, *J* = 0.6 Hz, 1H, H-2′); 7.41 (m, 2H, H-2′′ and H-6′′); 7.36 (m, 1H, H-6′); 7.27 (m, 1H, H-5′); 6.98 (tt, *J* = 8.4 and 2.3 Hz, 1H, H-4′′); 5.03 (ddd, *J* = 8.6, 5.1 and 0.7 Hz, 1H, H-1); 3.77 (m, 4H, H-3′′′′ and H-5′′′′); 2.76 (m, 2H, H-2a′′′′ and H-6a′′′′); 2.71 (s, 1H, H-2a); 2.70 (d, *J*= 3.9 Hz, 1H, H-2b); 2.50 (m, 2H, H-2b′′′′ and H-6b′′′′). ^13^C-NMR δ (ppm): 163.1 (dd, *J* = 255.8 and 11.7 Hz, 2C); 141.4 (t, *J* = 8.5 Hz, 1C), 135.8, 129.4, 125.8, 124.9, 124.2, 123.0, 121.0, 114.1, 111.0 (m, 2C), 110.0 (t, *J* = 25.0 Hz, 1C), 67.4 (2C), 64.8, 63.3, 54.0 (2C). Elemental analysis for C_20_H_20_F_2_N_2_O_4_S (422.45 g/mol) calcd.: C: 56.86; H: 4.77; N: 6.63; S: 7.59 Found: C: 56.69; H: 4.93; N: 6.74; S: 7.52.

### 4.4. ^1^H-NMR pK_a_ Determination Studies

All NMR spectra were performed on a Bruker Avance III HD-400 MHz spectrometer at 298 K. The ^1^H-NMR experiments were acquired using water suppression in order to suppress the residual HDO signal. Solutions of the **3b** and **4g** 2 mM in D*_2_*O were prepared adding acetone 200 μM as an internal standard [56]. The titrations were carried out progressively in a single NMR sample for each compound. The pH was adjusted with 0.1 mol/L of NaOH and 0.1 mol/L HCl, to cover the pH ranges 4.65–9.11 and 5.58–9.46 for **3b** and **4g** solutions, respectively. The pH ranges were chosen near to p*K*_a_ predicted by software MarvinSketch 16.5.2.0 from ChemAxon package [57,58]. The chemical shifts (δ ppm) of the piperazine methylene protons were plotted against the pH for both compounds. The p*K*_a_ were obtained from the inflection point of the resulting sigmoidal curve, for each compound.

**Figure 7:**
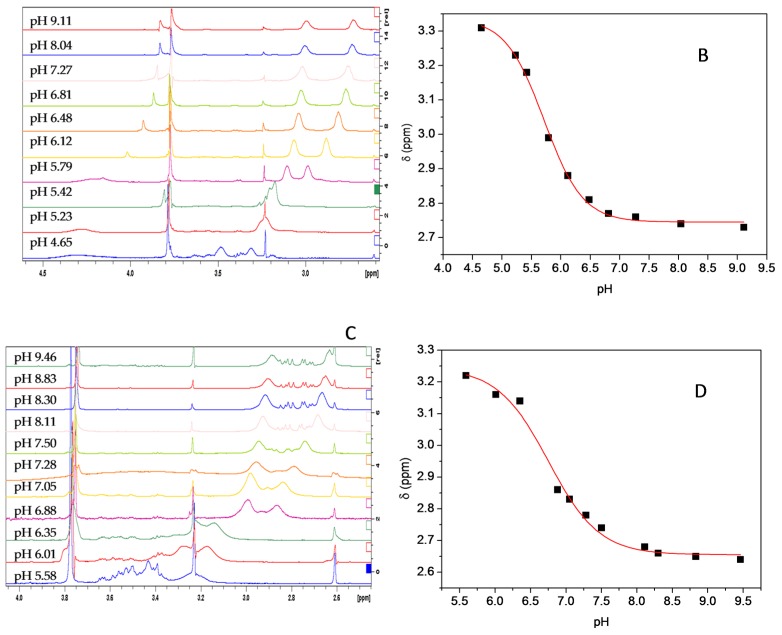
^1^H chemical shift dependence on the pH for compound **3b** (**A**,**B**) and compound **4g** (**C**,**D**). The biggest doublet corresponds to the four protons in the methylenes in the α-position to nitrogen in the piperazine. The p*K*_a_^D^ (p*K*_a2_) values are obtained from the inflection point of the resulting sigmoidal curve.

Figure 7 shows the ^1^H chemical shift dependence on the pH for the compounds **3b** and **4g**, respectively. Figure 7A,C shows an expansion of spectra, in the area of interest, for **3b** and **4g** compounds at different pH. The signal to upfield of the doublet of methylene protons of the piperazine ring was used to plot δ ppm vs. pH. In this case, the p*K*_a_^D^ correspond to inflection point in the resulting sigmoidal curve from the plot δ ppm vs. pH, for each compound (Figure 7B,D). The p*K*_a_ obtained were 5.72 and 6.69 for compound **3b** and **4g**, respectively. The p*K*_a_ value determined in D_2_O was corrected for H_2_O using the Krezel et al. equation [59].

The chemical shift is affected by the chemical and, consequently, the magnetic environment. Therefore, the protonation of basic species such as the nitrogen atoms in piperazine ring induce important changes in the chemical environment in the adjacent proton groups. Thus, considering the plot δ ppm vs. pH for the signal corresponding to methylene protons adjacent to nitrogen atoms in piperazine ring, we determined the second p*K*_a_ (p*K*_a2_) for the compounds **3b** and **4g** (Figure 7B,D). The values of p*K*_a2_ were 5.72 and 6.69 for **3b** and **4g**, respectively. Unsurprisingly, the p*K*_a2_ values, for both compounds, are lower than typical piperazine groups. This is because the compounds are tertiary amines. This type of amines are less basic than a secondary amine [60], concomitantly, the inductive effect of the distinct substituents induces further lower p*K*_a_ values for both compounds. Additionally, the p*K*_a_ values predicted in-silico are close to experimental values. All results reinforce the idea that the compounds here reported are weakly basic.

### 4.5. Radioligand Binding Studies

Affinity of compounds at 5-HT_6_ receptors was evaluated using HEK-293 cells expressing human 5-HT_6_R using the iodinated specific radioligand [^125^I]-SB-258585 (4-iodo-*N*-[4-methoxy-3-(4-methyl-piperazin-1-yl)-phenyl]-benzenesulfonamide); *K*_d_ = 1.3 nM; 2200 Ci/mmol). Competitive inhibition assays were performed according to standard procedures, briefly detailed below.

Fractions of 45 μL of diluted 5-HT_6_ membrane preparation were incubated at 27 °C for 180 min with 25 μL of [^125^I]-SB-258585 (0.2 nM) and 25 μL of WGA PVT SPA beads (4 mg/mL), in the presence of increasing concentrations (10^−11^ to 10^−4^ M) of the competing drug (5 μL) or DMSO, in a final volume of 100 μL of assay buffer (50 mM Tris, 120 mM NaCl, pH 7.4). Non-specific binding was determined by radioligand binding in the presence of a saturating concentration of 100 μM of clozapine. Binding of [^125^I]-SB-258585 to 5-HT_6_ receptors directly correlates to an increase in signal that was read on a Perkin Elmer Topcount NXT HTS (PerkinElmer Inc., Waltham, MA, USA). All compounds were tested at eight concentrations in triplicate. Clozapine was used as an internal standard for comparison. Data generated was analyzed using GraphPad Prism version 7.0 (GraphPad Software Inc., La Jolla, CA, USA). A linear regression line of data points was plotted, from which the concentration of competing ligand which displaces 50% of the specific binding of the radioligand (IC_50_ value) was determined and the *K*_i_ value was calculated based upon the Cheng-Prusoff equation: *K*_i_ = IC_50_/(1 + L/*K*_d_) where L is the concentration of free radioligand used in the assay and *K*_d_ is the dissociation constant of the radioligand for the receptor.

### 4.6. Pharmacological Profile Determination Assays

The pharmacologic profile of compounds was determined through an intracellular calcium mobilization assay [35] using a cloned human 5-HT_6_-expressing cell line (HTS111RTA, Millipore, Temecula, CA, USA) according to the manufacturer′s instructions with slight modifications. Immediately upon receipt, cells were placed in liquid nitrogen. Cells were thawed rapidly by removing from liquid nitrogen and immediately immersing in a 37 °C water bath. Immediately after the ice thawed, the exterior of the vial was sterilized with 70% ethanol. One milliliter of pre-warmed Media Component was added to each vial of cells (Media Component was supplied along with 5-HT_6_ cells). Contents from two vials were placed into a 15 mL conical tube and the volume brought to 10 mL with Media Component. The cell suspension was centrifuged at 190× *g* for 4 min. Supernatant was removed and 10.5 mL of pre-warmed Media Component was added to resuspend the cell pellet. The cell suspension was seeded in black, clear-bottomed 96-well plates at a density of 50,000 cells in 100 μL volume per well. Plates were incubated at 37 °C and 5% CO_2_ in an incubator overnight. Next day, the cells were loaded with Calcium 5 dye (R8186, Molecular Devices, Sunnyvale, CA, USA. Calcium 5 dye was made up according to the manufacturer′s instructions in HEPES-buffered Hank′s Balanced Salt Solution (HBSS) containing 5 mM probenecid at pH 7.4. Dye solution (90 μL) was added to the wells and incubated at 37 °C for 1 h. The plates were then placed in the Flexstation whereupon 10 μL of test compound or DMSO control was added and the fluorescence (ex/em: 485/525 nm) monitored to determine whether the compounds were acting as agonists. The compounds were tested with a final concentration of 0.1% DMSO. Three minutes after addition of compounds the Flexstation added 50 μL of agonist 2-Me 5-HT at a final concentration of 500 nM and the fluorescence (ex/em: 485/525 nm) was monitored to determine whether the compounds were acting as antagonists. Dose-response curves and IC_50_/EC_50_ values were generated using GraphPad Prism version 7.0 (GraphPad Software Inc., La Jolla, CA, USA). Values are the mean of duplicate data points. Error bars indicate standard error of the mean (SEM).

## 5. Conclusions

In conclusion, we present the design, synthesis, and biological evaluation of novel extended *N*-arylsulfonylindole derivative compounds as antagonists of the 5-HT_6_ receptor. A convenient synthesis of the extended arylpiperazine derivatives was achieved to readily access diversely substituted analogues. Several of the tested compounds exhibited nanomolar affinity for the 5-HT_6_ receptor. Finally, two compounds 1-(1-(4-iodophenylsulfonyl)-1*H*-indol-3-yl)-2-(4-(2-methoxyphenyl)piperazin-1-yl)ethanol (**4g**) and 2-(4-(2-methoxyphenyl)piperazin-1-yl)-1-(1-(naphthalen-1-ylsulfonyl)-1*H*-indol-3-yl)ethanol (**4j**) showed strong inhibition of 2-Me-5HT-induced Ca^2+^ mobilization in a cell-based assay, suggesting that potent cellular activity may be induced through antagonism of the 5-HT_6_ receptor.

## Figures and Tables

**Table 1:**
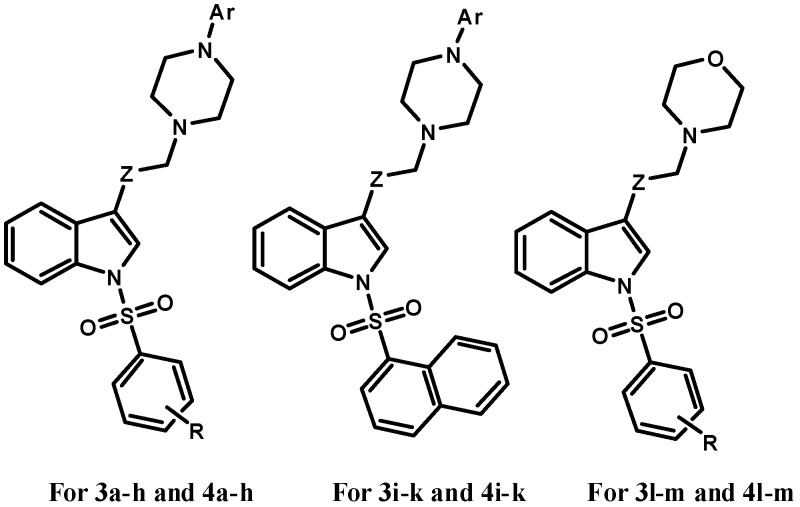
Yield and melting point data of new *N*-arylsulfonylindole derivatives ^a^.

Code	Z	R	Ar ^d^	Yield ^b^ (%)	m.p. (°C)
**3a**	CO	4-Me	2-Py	46	75–76
**3b**	CO	4-Me	2-MeOPh	66	132–134
**3c**	CO	4-Me	2-Pyrim	91	133–134
**3d**	CO	4-Cl	2-Pyrim	89	168–169
**3e**	CO	4-F	2-Pyrim	77	145–146
**3f**	CO	4-I	2-Py	93	179–180
**3g**	CO	4-I	2-MeOPh	98	182–183
**3h**	CO	4-I	2-Pyrim	96	184–185
**3i**	CO	-	2-Py	86	76–77
**3j**	CO	-	2-MeOPh	74	74–75
**3k**	CO	-	2-Pyrim	46	88–89
**3l**	CO	4-OMe	-	81	nd ^c^
**3m**	CO	3,5-diF	-	91	nd ^c^
**4a**	CHOH	4-Me	2-Py	98	72–74
**4b**	CHOH	4-Me	2-MeOPh	99	86–87
**4c**	CHOH	4-Me	2-Pyrim	68	81–82
**4d**	CHOH	4-Cl	2-Pyrim	75	76–77
**4e**	CHOH	4-F	2-Pyrim	69	72–73
**4f**	CHOH	4-I	2-Py	56	157–158
**4g**	CHOH	4-I	2-MeOPh	62	109–110
**4h**	CHOH	4-I	2-Pyrim	67	171–172
**4i**	CHOH	-	2-Py	72	80–81
**4j**	CHOH	-	2-MeOPh	61	89–90
**4k**	CHOH	-	2-Pyrim	65	82–83
**4l**	CHOH	4-OMePh	-	53	149–152
**4m**	CHOH	3,5-diFPh	-	66	nd ^c^

^a^ The products were characterized by IR and NMR spectroscopy and physical data. ^b^ Yields refer to pure isolated products. ^c^ Compounds in gel state. ^d^ Ph = Phenyl; Py = Pyridine; Pyrim = Pyrimidine.

**Table 2:**
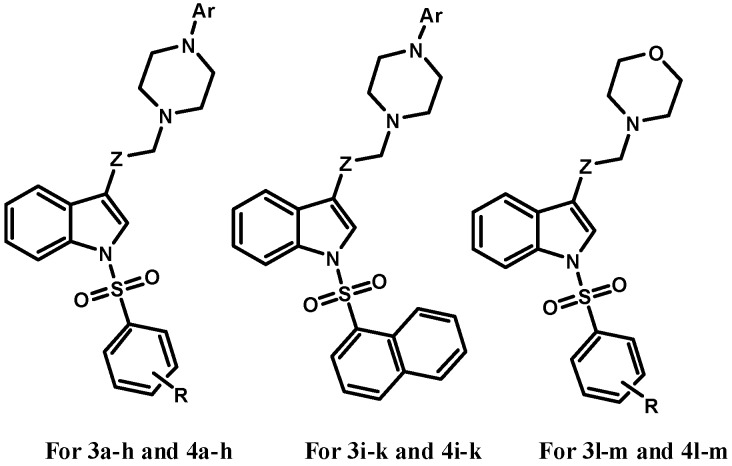
5-HT_6_R binding affinity results for new *N*-arylsulfonylindole derivatives ^a^.

Code	Z	R	Ar ^c^	p*K*_i_^b^	Code	Z	R	Ar	p*K*_i_^b^
**3a**	CO	4-Me	2-Py	5.75 ± 0.14	**4a**	CHOH	4-Me	2-Py	6.32 ± 0.19
**3b**	CO	4-Me	2-MeOPh	4.97 ± 0.18	**4b**	CHOH	4-Me	2-MeOPh	7.87 ± 0.15
**3c**	CO	4-Me	2-Pyrim	6.11 ± 0.14	**4c**	CHOH	4-Me	2-Pyrim	6.07 ± 0.15
**3d**	CO	4-Cl	2-Pyrim	5.07 ± 0.14	**4d**	CHOH	4-Cl	2-Pyrim	5.43 ± 0.20
**3e**	CO	4-F	2-Pyrim	4.72 ± 0.14	**4e**	CHOH	4-F	2-Pyrim	5.66 ± 0.20
**3f**	CO	4-I	2-Py	6.10 ± 0.08	**4f**	CHOH	4-I	2-Py	6.43 ± 0.15
**3g**	CO	4-I	2-MeOPh	5.72 ± 0.40	**4g**	CHOH	4-I	2-MeOPh	7.73 ± 0.09
**3h**	CO	4-I	2-Pyrim	6.38 ± 0.23	**4h**	CHOH	4-I	2-Pyrim	6.21 ± 0.21
**3i**	CO	-	2-Py	5.91 ± 0.14	**4i**	CHOH	-	2-Py	6.83 ± 0.14
**3j**	CO	-	2-MeOPh	5.90 ± 0.15	**4j**	CHOH	-	2-MeOPh	7.83 ± 0.14
**3k**	CO	-	2-Pyrim	5.85 ± 0.20	**4k**	CHOH	-	2-Pyrim	6.22 ± 0.19
**3l**	CO	4-OMe	–	6.35 ± 0.10	**4l**	CHOH	4-OMe	–	6.05 ± 0.07
**3m**	CO	3,5-diF	–	4.65 ± 0.11	**4m**	CHOH	3,5-diF	–	5.12 ± 0.15
**Clozapine**	-	-	-	7.92 ± 0.13	**1**	-	-	-	8.70 [1]
**5-HT**	-	-	-	7.12 [10]					

^a^ Displacement of [^125^I]-SB-258585 bound to cloned 5-HT_6_ human receptors stably expressed in HEK-293 cells. ^b^ IC_50_ values (not shown) were determined in triplicate and *K*_i_ values were calculated using the Cheng-Prusoff equation [33]. *K*_i_ = inhibitory constant from radioligand binding assays. In this assay, the IC_50_ value of clozapine was 12.4 nM; *K*_i_ = 11.9 nM, p*K*_i_ = 7.92. ^c^ Ph = Phenyl; Py = Pyridine; Pyrim = Pyrimidine.

**Table 3:**
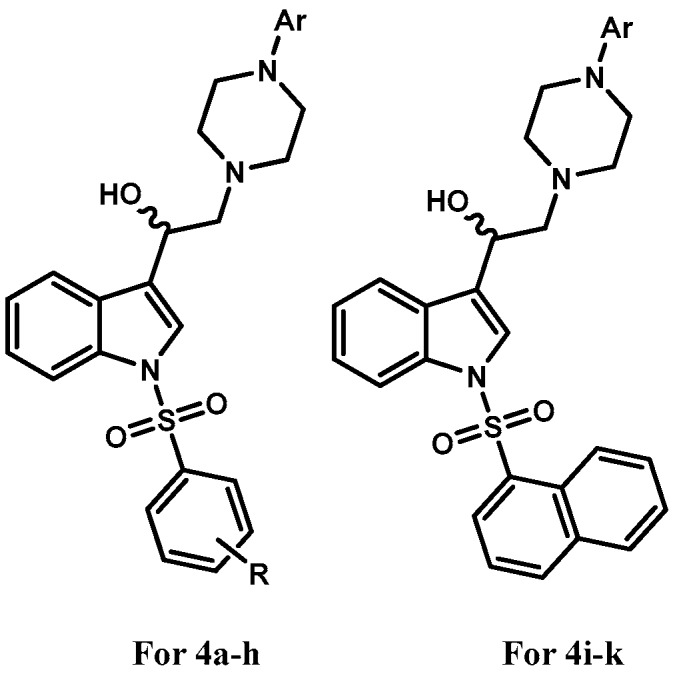
Antagonism of intracellular calcium mobilization **^a^**.

Code	R	Ar ^d^	IC_50_ (nM) ^b^
**4a**	4-Me	2-Py	783
**4b**	4-Me	2-MeOPh	nd ^c^
**4f**	4-I	2-Py	24,600
**4g**	4-I	2-MeOPh	204
**4h**	4-I	2-Pyrim	75
**4i**	-	2-Py	399
**4j**	-	2-MeOPh	32
**4k**	-	2-Pyrim	688
**Clozapine**	-	-	15.9

^a^ Antagonism of 2-Me-5-HT intracellular calcium mobilization assay. ^b^ IC_50_ values were determined using a non-radioactive cell-based assay [35]. IC_50_ values were determined in triplicate. In this assay, 2-Me-5HT′s EC_50_ value as agonist was 119 nM. ^c^ Not determined. ^d^ Ph = Phenyl; Py = Pyridine; Pyrim = Pyrimidine.

**Table 4:** Characteristics of identified binding sites based on cavities.

Site	X	Y	Z	Threshold	Volume (Å^3^)	Point Count *
1	−31.178	6.651	7.495	2.5	586.250	4690
2	−35.928	35.651	7.995	2.5	248.625	1989
3	−34.678	19.151	7.995	2.5	48.750	390
4	−26.678	50.151	12.995	2.5	30.875	247
5	−20.678	10.151	12.995	2.5	29.750	238
6	−29.928	24.901	0.245	2.5	23.375	187
7	−25.928	23.651	11.745	2.5	21.875	175
8	−20.428	42.901	−1.255	2.5	14.000	112
9	−44.678	26.901	10.245	2.5	13.250	106
10	−32.178	30.651	−2.755	2.5	12.875	103

* With a grid size of 2.5 Å.

**Table 5:** FRED docking scores (Chemgauss4) and other scoring functions for synthesized compounds.

Name	Index	p*K*i	ChemGauss4	LigScore1	LigScore2	PLP1	PLP2	Jain	PMF	PMF04	Ludi_1	Ludi_2	Ludi_3	ΔG Ludi_1	ΔG Ludi_2	ΔG Ludi_3	Consensus
3j	10	5.9	−19.57	3.5	4.72	139.88	140.26	6.05	579.9	546.47	810	687	1078	−11.05	−9.37	−14.7	11
4i_r	30	6.83	−19.36	4.71	6.25	136.12	132.47	5.99	558.69	533.96	705	615	1006	−9.61	−8.39	−13.72	11
4j_s	33	7.83	−19.2	4.15	5.34	140.77	142.09	5.92	613.33	583.74	826	717	1118	−11.26	−9.78	−15.25	11
3i	9	5.91	−19.06	3.25	4	125.45	122.41	6.49	575.64	532.3	748	632	921	−10.2	−8.62	−12.56	11
4j_r	32	7.83	−18.74	4.24	5.38	137.88	139.38	6.94	609.55	586.04	806	672	955	−10.99	−9.16	−13.02	11
4i_s	31	6.83	−18.69	1.13	0.91	131.81	131.06	5.65	549.81	530.82	683	605	1006	−9.31	−8.25	−13.72	11
3k	11	5.85	−18.58	3.78	5.17	127.85	124.86	5	536.9	515.62	756	643	1107	−10.31	−8.77	−15.1	11
4k_s	35	6.22	−18.22	3.04	4.05	126.09	123.73	5.62	547.04	530.75	633	569	875	−8.63	−7.76	−11.93	11
4k_r	34	6.22	−17.71	2.45	2.6	128.65	124.28	5.59	574.21	554.39	724	625	976	−9.87	−8.52	−13.31	11
3b	2	4.97	−16.92	−3.23	−6.13	98.92	108.88	7.62	549.29	521.19	778	657	1062	−10.61	−8.96	−14.48	9
3a	1	5.75	−16.73	−0.78	−1.85	106.66	109.34	4.59	520.04	492.33	812	644	1099	−11.07	−8.78	−14.99	11
4b_r	16	7.87	−16.52	−10.8	−17.97	85.74	100.29	5.15	542.51	531.71	781	678	1247	−10.65	−9.25	−17.01	8
4b_s	17	7.87	−16.52	−10.8	−17.97	85.74	100.29	5.15	542.51	531.71	781	678	1247	−10.65	−9.25	−17.01	8
4e_s	23	5.66	−16.02	2.2	2.38	119.97	115.27	5.14	486.2	494.54	546	528	878	−7.45	−7.2	−11.97	10
4a_r	14	6.32	−15.83	4.04	5.39	110.42	110.89	5.5	564.73	546.06	654	564	839	−8.92	−7.69	−11.44	11
4c_s	19	6.07	−15.62	1.94	2.08	117.91	116.9	4.88	501.32	507.06	585	526	880	−7.98	−7.17	−12	10
4c_r	18	6.07	−15.21	1.4	1.03	115.91	111.3	5.13	538.29	530.29	633	562	805	−8.63	−7.66	−10.98	11
3m	13	4.65	−15.17	3.43	4.5	104.81	101.28	4.67	353.06	373.45	533	494	697	−7.27	−6.74	−9.5	6
4m_s	39	5.12	−15.14	3.79	4.93	99.87	103.34	4.52	420.98	438.57	501	497	756	−6.83	−6.78	−10.31	5
4e_r	22	5.66	−15.02	2.74	3.57	111.75	103.89	4.76	505.89	512.95	597	539	770	−8.14	−7.35	−10.5	11
4g_r	26	7.73	−14.89	−20.63	−34.04	107.72	112.42	5.82	513.78	514.57	719	659	1054	−9.8	−8.99	−14.37	9
4f_r	24	6.43	−14.83	−3.18	−6.58	108.34	112.04	5.56	517.06	509.01	689	598	887	−9.4	−8.15	−12.1	9
3g	7	5.72	−14.68	−7.37	−10.74	78.7	75.52	5.54	450.15	434.06	637	589	937	−8.69	−8.03	−12.78	5
4d_r	20	5.43	−14.61	3.32	3.88	107.75	105.26	5.85	511.12	504.56	645	560	856	−8.8	−7.64	−11.67	11
3f	6	6.1	−14.5	1.12	2.88	93.3	91.41	4.69	425.98	412.79	590	520	852	−8.05	−7.09	−11.62	6
4a_s	15	6.32	−14.42	−1.95	−3.57	103.05	104.18	4.43	525.34	513.3	615	573	847	−8.39	−7.81	−11.55	8
4g_s	27	7.73	−14.33	0.51	1.64	97.13	95	5.79	507.06	491.66	654	575	905	−8.92	−7.84	−12.34	10
4m_r	38	5.12	−14.22	−0.29	−1.12	93.7	91.7	4.12	421.32	437.9	435	444	648	−5.93	−6.05	−8.84	3
4d_s	21	5.43	−14.07	2.62	2.71	103.82	102.47	4.15	495.37	480.52	606	510	745	−8.26	−6.95	−10.16	8
3c	3	6.11	−14.04	2.45	3.16	98.42	96.69	4.61	530.61	510.38	726	612	886	−9.9	−8.35	−12.08	11
4h_s	29	6.21	−13.9	−20.04	−33.18	102.75	112.55	5.45	463.4	471.58	642	590	942	−8.75	−8.05	−12.85	8
4h_r	28	6.21	−13.87	1.06	0.61	105.96	101.81	4.83	518.85	512.81	633	554	847	−8.63	−7.55	−11.55	10
3e	5	4.72	−13.86	1.65	1.18	97.36	103.7	5.89	467.09	474	687	597	1056	−9.37	−8.14	−14.4	10
4f_s	25	6.43	−13.77	−3.61	−5.79	92.37	90.26	4.54	459.24	453.03	584	534	811	−7.96	−7.28	−11.06	3
4l_s	37	6.05	−13.65	2.96	4.15	101.24	99.41	4.39	475.3	478.99	509	481	681	−6.94	−6.56	−9.29	6
3d	4	5.07	−13.55	3.07	3.55	105.47	98.38	4.03	487.82	466.13	717	593	751	−9.78	−8.09	−10.24	8
3l	12	6.35	−13.45	3.55	4.73	93.97	90.15	4.49	494.34	467.72	590	498	679	−8.05	−6.79	−9.26	6
4l_r	36	6.05	−13.03	−2.86	−5.51	92.12	92.12	4.03	466.35	460.71	509	481	695	−6.94	−6.56	−9.48	1
3h	8	6.38	−12.46	−1.04	−1.54	92.3	88.74	5.12	433.2	419.93	631	555	891	−8.6	−7.57	−12.15	6

ΔG binding from Ludi Scores calculated as ΔG = LudiScore/−73.33, in kcal/mol.
